# Potential Role of Nanoparticles in Treating the Accumulation of Amyloid-Beta Peptide in Alzheimer’s Patients

**DOI:** 10.3390/polym13071051

**Published:** 2021-03-27

**Authors:** Mohamed Abbas

**Affiliations:** 1 Department of Electrical Engineering, College of Engineering, King Khalid University, Abha 61421, Saudi Arabia; mabas@kku.edu.sa; 2 Department of Computers and Communications, College of Engineering, Delta University for Science and Technology, Gamasa 35712, Egypt

**Keywords:** nanodrugs, Alzheimer’s, amyloid beta-peptide, nanoparticles, lipids, drug delivery

## Abstract

The disorder of Alzheimer’s is marked by progressive pathophysiological neurodegeneration. The amino acid peptides in the amyloid plaques found in the brains of people with Alzheimer’s disease (AD) are known as amyloid-beta (Aβ). Current treatments are not curative, and the effects associated with AD are reduced. Improving treatment results involved the targeting of drugs at optimum therapeutic concentration. Nanotechnology is seen as an unconventional, modern technology that plays a key role in the treatment of Alzheimer’s disease. Using nanoparticles, molecular detection, effective drug targeting, and their combination offer high sensitivity. The aim of this review is to shed light on the function and successful role of nanoparticles to resolve Aβ aggregation and thus to help cure Alzheimer’s disease. The analysis divides these nanoparticles into three categories: polymer, lipid, and gold nanoparticles. A thorough comparison was then made between the nanoparticles, which are used according to their role, properties, and size in the procedure. The nanoparticles can prevent the accumulation of Aβ during the efficient delivery of the drug to the cells to treat Alzheimer’s disease. Furthermore, this comparison demonstrated the ability of these nanoparticles to deal efficiently with Alzheimer’s disease. The role of these nanoparticles varied from delivering the drug to brain cells to dealing with the disease-causing peptide.

## 1. Introduction

**Figure 1:**
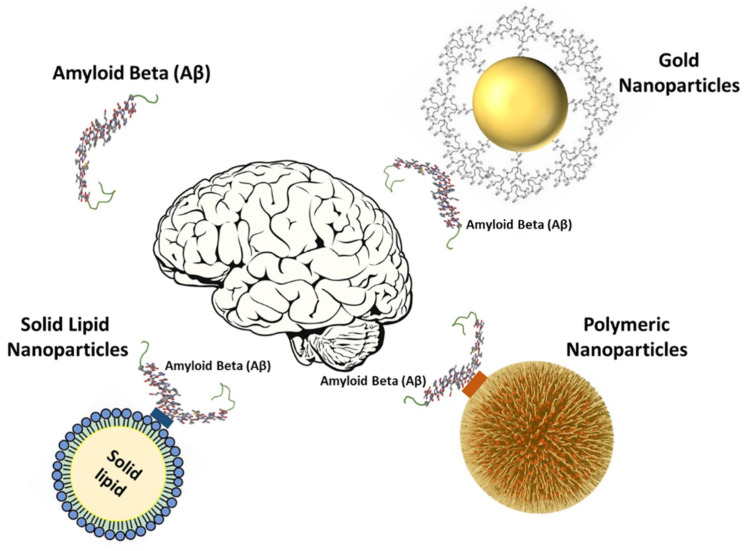
Effects of different types of nanoparticles on the treatment of Alzheimer’s disease (AD).

Alzheimer’s Disease (AD) is the induction of cognitive and conducted impairment in neurological disorders. Conventional therapeutic approaches, including inhibitors of acetylcholine-esterase, often fail due to insufficient solubility, decreased bioavailability and a lack of obstacles to the brain and blood. This disease, one of the biggest global healthcare problems, is the most common form of dementia. It is a neurodegenerative disease causing progressive cognitive performance and memory loss. A potential field of research for the treatment of AD was recently identified by nanotechnology. Furthermore, it is one of the oldest diseases, and fewer than 5% of AD cases are inherited directly, and so environmental factors may play an important role in the initiation and advance of the disease. Nanotechnological approaches to care include design, characterization, development, and implementation of clinical enhancement nano-systems. Nanoparticles are composed of polymers of nanoparticles, lipid compounds, fluids, liquid crystals, and nanos. Nanoparticles All are promising instruments for the supply by various routes of administration of therapeutic equipment like intranasal treatment [1]. The accumulation of Aβ in the brain would contribute to pathogenic AD cascades. Although beta-amyloid formation appears to be located primarily in neurons, it exists in a wide variety of molecular forms. Amyl precursor peptide is created when the smaller protein called amylase begins to break down. A type of allergenic amylase is suspected to be the cause of causing multiple sclerosis. Although amyloid clumps occur naturally and impair cell function, levels of amyloid precursor protein interaction, there is not sufficient in the brain to cause the disease, resulting in its initial development. The strategy for Aβ clearance is an active way to study it as a possible cure for the modification of diseases [2]. A 29 amino-acid peptide derived from the rabies virus glycoprotein (RVG29) was used to target the brain and to enhance the neuronal nicotinic acetylcholine receptors (nAChRs) absorption. To take advantage of its neuroprotective advantages [3], these nanosystems were combined with quercetin, especially for Alzheimer’s disease. Because of twisted protein binding microtubular strands of amyloid fibril in the brain. The treatment of AD includes hundreds of smaller molecular inhibitors. The blood–brain barrier (BBB) [4] cannot in any way block all these medicines. The blood–brain barrier does not exist. Nerve cells in the brain neurons are expected to accumulate Aβ peptides in Alzheimer’s disease and to lead to progressive memory loss. There are signs of initiation and development of AD because of Aβ changes, especially the generation of neurotoxic oligomers 10–20 years before deficiency [5]. The functional cognitive disorder may also occur well before the disease begins if Aβ is produced and cholinergic systems dislocate. Nanoparticles combine the targets, visualization, and treatment in one form to give drug molecules new hope and cross BBB [6]. Figure 1 shows the effects of different types of nanoparticles on the treatment of Alzheimer’s.

Because the BBB level with nanoparticles is difficult to cross, particles will cross the BBB within the 50–100 nm range without complex adaptation. If there is a limit to the crossing of the BBB [7], a therapeutic solution is recommended for each nanoparticle derivative. The extra iron dramatically accelerates AD in the brain, and the cells are destroyed by excess iron. On AD plates and in situ-considered encounters because of pathological iron dysfunction, magnet nanoparticles able to catalyze the formation of reactive oxygen species [8] are present. To avoid and minimize the growth of Aß, combination therapy is required to simultaneously manage the imbalance of acetylcholine as a potential cure for AD. Clioquinol (metal-ion chelating agent) and donepezil (acetylcholinesterase (AChE) inhibitor) co-encapsulated human serum albumin (HSA) nanoparticles (dcHGT NPs) [9]. A central limiting factor in brain supply is the BBB. The intention was not to concentrate on pharmaceuticals using traditional medicinal products. The most promising mechanism in the delivery of nasal drugs was an improvement in brain medicine [10]. Pharmaceutical products availability. Lower solubility, low blood–brain barrier and low dryness of anti-AD products decrease therapeutic efficacy.

In this connection, large and small molecular medicine for the treatment of AD seems to be promising. Nasal supplies are a popular road. This promising trip results in bad neighborhood systemic results, greater bioavailability, and therapeutic efficacy [11] on the olfactory route to the brain. The primary Aβ fibrillation engine is the Lys-Leu-Val-Phe-Phe (KLVFF) series. Aβ is also used for attack, and Aβ aggregation can be avoided [12]. Based on significant nanoscience and nanotechnology advancement, biosensor advances have made significant progress in recognizing essential AD biomarkers. The special and special features of nanomaterials improve the electrochemical and optical activity of the transducer to immobilize biological components of detection [13]. The aim of this review is to shed light on the use of nanotechnology in treating Alzheimer’s disease using nanoparticles. To clarify the effective role of these nanoparticles, they were divided into three sections, and they are polymeric, lipid and gold nanoparticles. The next sections will deal with more details about them. A comparison is made between the characteristics of each and the volumes used in each of the treatment methodologies.

## 2. Polymeric Nanoparticles Effects on Amyloid Beta Peptide

Diseases to cure multiple diseases and to resolve treatment hurdles promise to be treated easily with multifunction. The main enzyme in Aβ formation has been reduced by the supply of non-coding plasmid ribonucleic acid (RNA). Concurrent delivery of therapeutic peptides to the brain helps to reduce neurofibrillary entanglements [14]. It has been extensively explored on the routes of nano-sufficiency, such as liposomes, polymeric nanoparticles, micelles, conjugates, peptide carriers, cyclodextrins, stable dispersions, lipid nanoparticles, and emulsions. The effect of molybdenum disulfide inhibition is known as molybdenum disulfide (MoS2). A laser-pulsed removal method was used in polyvinylpyrrolidone functional MoS2 NPs.

In Aβ aggregation inhibitors, Aß destabilization, oxidative stress relaxation caused by Aβ and cell toxicity, multifunctional effects have been observed. Initially, MoS2 NPs obstructed the production of cell membrane channels [15] triggered by Aβ-fibrillation. In addition to nerve growth factor (NGF), release and disease modification approaches to AD, the role of nanoparticles in the brain, in genes, and in cell screening therapy from polymerized implants, as a neuroprotector, was emphasized. Promising nanoparticles and quantum points, lipids, and polymer-dependent delivery mechanisms have been investigated to achieve the present NGF therapeutic weaknesses [16]. Deposits are disposed of to shield them from Aβ. While several aggregation inhibitors have been studied, there are only limited NP ratios. NPs provide an ideal environment for tunable Aβ-rational structure, surface, and size aggregation inhibitors. The degree of aggregation was modified by the NP and surface chemistry, while the aggregate morphology was determined by the electrical charge of the NP. The mixture of Aß was repealed at 8 nm and 18 nm of poly-coated NPs (acrylic acid), with a sub-stoichiometric ratio of 1:2,000,000 [17]. Aβ 1-42 model tested the neuroprotective efficacy of anthocyanin-powered nanoparticles polyethylene glycol-gold (PEG-AuNPs).

Both mice were Aβ1-42-injected with PEG-AuNP primed anthocyanin and anthocyanin with increased memory defects (12 mg/g/d 14 days). The use of Aß1-42 mice of PEG-AuNPs inhibited anthocyanin was reported [18]. Nano conjugates were also differentiated by spectrophotometric absorption, dynamic illumination, and electron microscopy, among other methods. We find that it does not affect the viability of neuronal nanoconjugation; it penetrates cells and decreases Aß peptide in vitro aggregation. The toxicity of aggregated Aβ peptide was also decreased in the *Caenorhabditis elegans* AD model [19]. A gold nanoparticle polyoxometalate with Wells–Dawson structure peptide (AuNPs@POMD-pep) has the synergistic effect of inhibiting Aβ aggregation, dissociating Aβ-fibril, and reducing cytotoxicity through mediated peroxidase Aβ activities. Using AuNPs@POMD-pep, the use of BBB to deal with drawbacks of small molecular anti-AD medication [20] is being used to cross BBBs. Approved expression and successful clearing in microglia and liver cells of the Aß low-density lipoprotein receptor (LDLR) α-mangostin, which is in vivo is decreased due to hydrophobia, low solubility and aqueous environ-mental stability, hence low bioavailability and objective aggregation of bacteria. PEG-PLA was encapsulated to overcome this limitation. To overcome this limit, poly-metals were encapsulated [21]. Carbon points (CDs), which were embraced using Eu/GMP infinite coordination polymer (ICP) self-adapting chemistry with large functional groups, could not only be used to effectively sensitize the red fluorescence of Eu/GMPIs as a personal guide to self-correction. The CDs@Eu/GMP ICPs were produced, while Cupric ion Cu + 2 emissions were missing. They included the CDs 400 nm and 592 nm, 615 nm, 650, and 694 nm. The emissions of StrongEu3 + were noted. The combination of CD and Cu + 2 diminished, creating a damaging antenna effect because of the addition of Cu + 2, the red fluorescence of europium (Eu3 + ) decreased. After the addition of Aβ monomer and Eu3+ red fluorescence, the special bonding between Cu + 2 and Aβ monomer has been restored. To track internal changes in the atmosphere and to detect Aβ monomers in bio-logical fluids, the fluorescence of CDs remained the same in this process [22]. The RVG29 nanoparticles were less than 250 nm spherical in size and brain applications [23].

Alteration of poly-lactide-co-glycolides and selenium nanoparticles’ encapsulation in Alzheimer’s disease therapy can improve bioactivity and drug delivery characteristics of curcumin nanoformulation (Se NPs). It has been examined using analytical instrument techniques to determine the moral structures of the polymer, the distribution of the scale of the nanosphere, and the chemical interactions between the polymer and the synthesized curcumin nanoformulation. The first nanoparticles of protein-coated metal (PC) were examined in vitro to inhibit tau. New features of iron oxide of ferrimagnetic magnetite protein-capped (PC-Fe3O4) and protein-capped (PC)-cadmium sulfide (CdS) nanoparticles have been presented as active TAU aggregation inhibitors of spectrometry, poly-acrylamide sodium sulfate electrophoresis and electron microscopy. Nanoparticles with biologically synthesized PC metal, particularly iron oxide, do not affect the viability of neuroblastoma cells. Furthermore, PC-CdS nanoparticles have double properties for tau inhibition and breakdown. Nanoparticles can be used as potent tau aggregation Inhibitors and can be modified due to their relatively small size for drug delivery. To provide a fascinating insight into the role of biological nanostructure in the disease of Alzheimer’s, an unprecedented strategy for the design of anti-tau aggregation drugs was presented [24].

**Figure 2:**
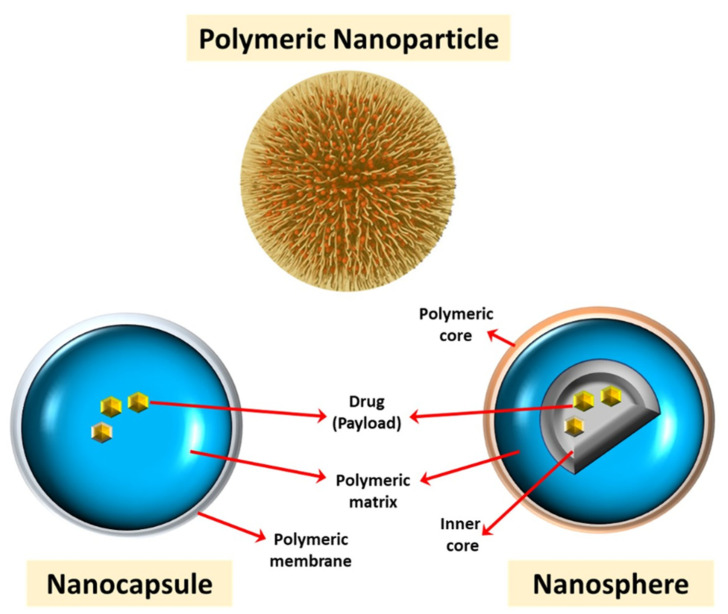
The role of polymeric nanoparticles on the treatment of AD.

The accumulation of amyloid plaques, neurofibrillary encounters, and other symptoms of AD may result from diabetes and obesity, including insulin resistance, hyperglycemia, hyperinsulinemia, chronic swelling, oxidative stress, adipokine dysregulation and vascular dysfunction. Currently, polyphenols have been increased in animal and in vitro models due to their relatively insignificant effects. Quercetin (QT) is one of the fruit and vegetables with a wide range of diseases and various bio-based and health-promoting effects, among the most abundant polyphenolic flavonoids. Researchers developed various QT-included nanoparticles to over-come these limits: low bioavailability and limited QT solubility nanoparticles. The key molecular pathways to increase AD pathogenesis caused by diabetes and obesity were addressed. Concave cubic quercetin-modified gold-palladium (Qu@*P*-80@AuPd) allows autophagy of human neuroblastoma (SH-SY5Y) cells, facilitates autophagosome and lysosome fusion, speeds Aβ clearance, and defends SH-SY5Y cells against cytotoxicity damage caused by Aβ. Concave cubic BBB is also highly permeable and biocompatible with Qu@*P*-80@AuPd [25]. Figure 2 shows the role of polymeric nanoparticles in the treatment of AD.

A natural compound, which binds directly to amyloid plates, is used in magnetic nanoparticles made from curcumin-conjugated super magnetic iron oxide (SPIO). Curcumin-conjugated magnetic nanoparticle coatings with polyethylene glycol-polylactic acid block copolymers are generated in a multi-insert vortex mixer by stable and biocompatible curcumin magnets with a mean anti-solvent precipitation of <100 nm. Via transmission electron microscopy and microscopic atomic force, the nanoparticles were also visualized, as were X-ray diffraction, thermogravimetric analysis, x-ray photoelectron spectroscopy, secondary ion mass spectrometry and Fourier-transform infrared spectroscopy. There was no cytotoxicity to curative MNPs in Madin-Darby renal canine (MDCK) or differentiated human neuroscience cells SH-SY5Y. A study of mouse immunohistochemistry indicated that amyloid pills were found along with curcumin-conjugated magnetic nanoparticles [26].

To improve sensitivity in AD diagnosis, there have been updated two fluorescein isothiocyanate-labeled peptides Aβ42 and the polyvalent-directed ultrasensitive peptide polymer (PDPP). The dissociation of Aβ42 for PDPP was 103 times the site-directed peptide. The enhanced binding was because the PDPP could detect multiple receptors on the target. The strength of the diagnostic PDPP probe was tested in the fg mL^−1^ range, which is more sensitive than the antibodies or single peptide detection when used in the detection of Aβ42 in cerebrospinal fluid (CSF). The feedback based on the properties of nanoporous zinc oxide (ZnO) nanoparticles has been increased, and Aβ42 has been calculated and quantified for an ultra-low concentration (ag-mL^−1^). The PDPP coupled with the ZnO-based nanoporous method constitutes a new approach to the diagnosis of AD, which can also help detect other target biomarkers and clinical applications [27]. The immunomagnetic capture of Aβ40 and Aßen 42 peptides with tau protein pumped up into CSF and serum imitating samples using suitable conjugated antibodies was investigated with anti-biofouling polymer polymers glycol block allyl glycidyl ether (PEG-b-AGE) covered magnet iron oxide nanoparticles (IONPs). In contrast to antibody-conjugated magnetic beads (Dyna beads, 50 percent specialty, and 30–40 percent sensitiveness), typically used as magnetic separators under the same experimental conditions as non-target interference proteins, anti-body conjugated IONPs showed an increased specificity (>90 percent) and sensitivity (>95 percent). The antibody-conjugated IONPs were also substantially higher than the antibody-conjugated Dyna beads (+20%) [28], with a substantial improvement in sensitivity (80–90%) and stronger capture of Aβ or tau protein from human whole blood samples. Nanocarriers were designed to: interact with Aβ1-42 in the blood and en-courage its removal through the “sink” effect; and correct memory defects found in transgenic AD-like mice. Surface-operated surface nanoparticles with Aβ1-42 antibodies were biodegradable and PEGylated. Treating the AD-like transgenic mice with the anti-Aβ1-42 functional nanoparticles resulted from the full memory correction defect; the level of the Aβ-soluble peptide and its brain oligomer was significantly reduced, and the plasma Aß levels increased significantly [29].

Mitochondria-focused nanozymes known as triphenyl phosphonate (3-carboxypropyl) bromide-functional molybdenum disulfide quantum points (TPP-MoS2 QDs) have been formed for the purposes of combination 1,2-diesteroyl-sn-glycero-3-phosphoethanolamine-N (amino (podiatylene glycol)-2000) [30]. An AD-pathological event expressed as cognitive dysfunction results in overproduction and accumulation of Aβ-peptide 1–42 (Aβ (1–42)). Ginsenoside Rg3 is a major ginseng component, which plays a key role in memory and enhanced knowledge and which, by reducing free radicals, induces antioxidant effects. In the treatment of AD as a neuroprotective agent, Ginsenoside Rg3 may be an excellent candidate. Biodegradable PLGA nanoparticles have been formulated and characterized as encapsulating ginsenosides Rg3 and thioflavin T, Aβ diagnoses; examining their neuroprotective effects; investigating key mechanisms that may underlie their neuroprotective effects and assessing their ability to cross BBB using the in vitro BBB model. PLGA-Rg3 are promising new therapeutic materials that can be used for the identification and treatment of AD [31] in natural nutraceuticals.

### 2.1. Role of PLGA in Treating AD as a Polymeric Material

A kind of poly-lactide-co-glycolic acid (PLGA) nanoparticles have been designed by loading with Aβ generation inhibitor S1 (PQVGHL peptide) and curcumin to target the detrimental factors in AD development and by conjugating with brain targeting peptide CRT (cyclic CRTIGPSVC peptide), an iron-mimic peptide that targets transferrin receptor (TfR), to improve BBB penetration. The median diameter of drugs in PLGA was 128.7 nm and 139.8 nm in calreticulin (CRT) PLGA. This type of nanoparticles inhibited the accumulation of Aβ, reactive oxygen species (ROS), tumor necrosis factor-alpha (TNF-α), and Interleukin 6 (IL-6) and raised the concentrations of superoxide dismutase (SOD) and synapse levels in the brains of AD mice [32]. In-plant models for polyethylene glycol (PEG), use of solvent displacement techniques was synthesized (PLGA-PEG). There are several proven experiments using human brain cell lines that protect neuronal cells against oxidation-induced apoptosis. The difficulty in delivering therapeutic proteins into the central nervous system is really a disadvantage of the protein [33].

PLGA-soya lecithin-Tween−80 nanoparticles have been synthesized in combination with self-assembly using revised nanoprecipitation techniques. The effects of substantial particle size, polydispersity, trap efficacy and drug release factors in vitro have been studied. The optimized formulation (D10) of prepared nanoparticles showed particle size 171.74 nm, polydispersity 0.154, trap efficacy 66.171 percent and drug release 67.336 ± 0.254 percent in vitro (60 h). Zeta six-month potential and stability tests have shown that the refrigerator formulations are stable (3–5 °C) and most suitable for nanoparticle storage [34]. For the generation of enhanced brain-encapsulated iAβ5 drugs, PLGA nanoparticles with monoclonal anti-personnel and antimonoclonal receptors have been used.

Porcine brain capillary endothelial cells (PBCECs) have been taken as a BBB model to assess the efficacy and toxicity of the device. Improvement in the absorption of immune nanoparticles with regulated peptide iAβ-5 compared to nanoparticles from non-monoclonal antibody functions was significant [35]. The vitamin D-binding protein (DBP) can be diminished and accumulated. Biocompatible polymers PLGA can be filled with therapeutic agents, and their release rates can be controlled. In this experiment, a PLGA-based drug delivery method was used to analyze the therapeutic effects of DBP-PLGA nanoparticles on Aβ-overexpressing (5XFAD) mice. The DBP has been loaded into PLGA, and DBP-PLGA nanoparticles’ properties have been analyzed. The aggregation of Aβ in vitro by a thioflavin-*t*-test by DBP-PLGA nanoparticles has been substantially preventable. Furthermore, the accumulation of Aβ, neuroinflammation, loss of neurons and cognitive dysfunction in mice with 5XFAD were substantially decreased by intravenous DBP-PLGA nanoparticle injection [36]. The findings of synthesized curcumin-loaded nanosphere microscopic and nano observations are proven to be mono-dispersive spherically shaped particle distributions. AD mice ‘new brain samples could decrease the Aβ load and significantly reduce the memory deficit of the model mice in the new drug delivery systems of curcumin-loaded selenium poly-lactide-co-glycolide (Se-PLGA) nanoparticles.

The technique of microscopic fluorescence visualized the exact relation of the Se-PLGA curcumin-charged nanoparticles to the plaques Aβ. In AD lesions with the transgenic mouse (5XFAD) tested, the Se-PLGA plate supply system may provide improved therapeutic effectiveness [37]. Memantine was used for the production and covering of biodegradable PLGA nanoparticles using a dual emulsion technique. Memantine polyethylene glycol poly lactide-co-glycolic acid (MEM-PEG-PLGA) nanoparticles were developed to treat Alzheimer’s disease by oral administration with a BBB. For the design of the experiment, output parameters were optimized. Mean particle size for MEM–PEG-PLGA nanoparticles exhibited below 200 nm, monomodal size distribution (polydispersion index, PI < 0.1) and adverse surface load (−22.4 mV), respectively.

Physicochemical characterization of these nanoparticles has shown the dispersion of the crystalline medicine within the PLGA matrix. MEM-PEG-PLGA NPs were found to be non-cytotoxic in the brain cell line (bEnd.3 and astrocytes). Memantine, which decreases the rate of drug use in vivo, followed the slowed-down profile of NPs against a free drug solution. In vitro and in vivo, nanoparticles have been able to cross BBB. The use of MEM-PEG-PLGA NPs has increased the advantage of reduced memory failure compared with the free drug solution. Histological studies have confirmed that MEM-PEG-PLGA nanoparticles have been reduced to Aβ plaques with associated inflammation of AD [38].

#### Combining Quercetin and PLGA as a Polymeric Material

Quercetin is significant for countering Alzheimer’s. It is a promising agent for Alzheimer’s because it is biocompatible, and it can penetrate the brain tissue. Nanoparticles were engineered to attack and eliminate tumors. It was concluded that nanoparticles existed because of FTIR and NMR. A variety of particle shapes were found in the experiments. The Hydrogel size is from 200 to 300 nm. It is found that 85% of quercetin molecules bind to the chalcones complex. More than 99% of the capillaries were filled with Aβ peptides, and the fibrillation rate of Aβ was reduced [39]. PLGA-functionalized quercetin (PLGA@QT) cytotoxicity experiments yielded increasing cytotoxicity by increasing concentration. The neurotoxicity of zinc-Aβ: the 42-residue (Aβ42) systems was inhibited by the PLGA-QT NPs. In vivo systemic toxicity of PLGA@QT NPs has been investigated in major organs through histological examination, and no evidence of adverse reactions has been identified in mice [40].

Nuclear magnetic resonance (NMR) and Fourier-transform infrared spectroscopy (FTIR) have been tested for practical use of rabies virus glycoprotein peptide (RVG29) peptide nanoparticles. These nanosystems were loaded with quercetin to take advantage of its neuroprotective properties, mainly for Alzheimer’s disease. The sample, polydization and zeta potential of nanoparticles have been measured with transmission microscopy, and the dynamic light distribution has been calculated. For the brain microvascular endothelial cell line (hCMEC/D3), a blood–brain barrier and thioflavin T-assembly model, in vitro research on the normal amyloid-beta fibrillation mechanism for Alzheimer’s was conducted. Then sum up in vitro, in vivo, and clinical proof QT anti-Alzheimer’s, antidiabetic, and anti-obesity effects [41]. Autophagic dysfunction was linked to AD pathogenesis. Autophagic activation is, therefore, a potential way to eliminate intracellular Aβ and reduce Aβ-induced neurotoxicity.

Because of anti-amyloid, anti-inflammatory, antioxidant, curcumin was shown to be potentially used for Alzheimer’s disease. However, their hydrophobic and poor bioavailability inhibit their use. The conjugate (PLGA-PEG) was developed with B6 peptide and packaged with curcumin (PLGA-PEG-B6/Cur), and provided for the transgenic mouse APP/PS1 and HT22 cells. In vitro experiments, including dynamic light scatter (DLS), flow cytometry (FCM), red blood cell analysis (RBC), thromboelastography (TEG), and DLS, have shown curcumin’s diameter can be decreased, cell uptake increased and consistent with the blood. The spatial learning and memory ability of APP/PS1 mice could significantly improve with PLGA-PEG-B6/Cur compared with the original Cur. Ex vivo studies have shown that PLGA-PEG-B6/Cur can minimize Aβ formation and tau hyperphosphorylation, including silver bleaching, immunostaining, and West-blotting [42].

### 2.2. Role of Pittsburgh Compound B (PiB) in Treating AD

Early diagnosis of AD is still complicated, including imagery. The relief rate of Mn0.6Zn0.4Fe2O4 (MZF) modified by PiB to specifically bind to amyloid plaques was 169.93 mM^−1^S^−1^. In both cell lines, there has been no cytotoxicity. The in vitro mixture of Aβ plaque with brain sections of a six-month-old AD mouse [43] has been shown by immune-histochemistry. In vitro synthesis and study of magnetic nanoparticles targeting β-amyloid panels is the Alzheimer’s biomarker. Using a transmission microscope, the morphological characteristics were examined in the MZF-PiB. The paramagnetism of the MZF-PiB is evaluated with 3 T MRI scanning r (2). For the determination of MZF-PiB nanoparticles, the cytotoxic analysis was carried out in differentiating human cells that are thrice-subcloned cell lines derived from the SK-N-SH neuroblastoma cell line (SH-SY5Y) and in canine kidney cells of Madin-Darby (MDCK) [44].

Despite using [11C]-PiB (Aβ), the other approved derivatives [18F] flutemetamol and stilbene ([18F]-florbetaben and [18F]-florbetapir) are now thoroughly investigated and advised for AD as soon as practicable despite the use of gold standard [11C]-PiB. Recently, a growing trend in research into the manufacture of PET-binding neurofibrillary tangles (NFTs) for monitoring disease development has been observed. Many tau positron emission tomography (PET) ligands have shown good affinity and tau pathology. There is no therapeutic response for this disorder, although there has been a comprehensive analysis of early detection and production of AD with PET imagery. Therapeutic methods of nanoparticles have appeared recently. In the reduction/inhibition of amyloid and tau hyperphosphorylation [45], the coordination of organic NP surface ligands and inorganic NP was investigated.

## 3. Lipid Nanoparticles Effects on Amyloid Beta Peptide

Both metabolites given intravenously can be quickly metabolized in the liver and the intestine into two dangerous metabolites. One easy way to remove toxins is by using combined subunits. That is not like an antidepressant as the substance cannot pass through the blood–brain barrier. The particles of surfactant were prepared by a double emulsion evaporation technique. Glaucoma is a disease in which the patient’s vision slowly reduces over the course of days or weeks, and it eventually goes completely. The two situations are often linked for many reasons. The particles also help protect the nerve, and especially the brain, by promoting the delivery of nanotechnology. Luca Technologies is one of the primary companies that master the field of drug delivery. It is possible that the medicines that are causing the disorder are made stronger through the help of nanoparticles [46]. The brain supply system Beta-Secretase 1 (BACE1) with small interfering RNA (siRNA) is optimal and functional. A short peptide extract from the chimeric rabies virus glycoprotein fragment (RVG-9R) glycoprotein virus enhances transcellular trajectory in neuronal cells. The perfect molar relationship between siRNA and BACE1 was thus demonstrated. The installation between them was screened. The nasal delivery system was proposed for olfactory and trigeminal pathways. The coating process affects the loading and protection of nanoparticles [47].

**Figure 3:**
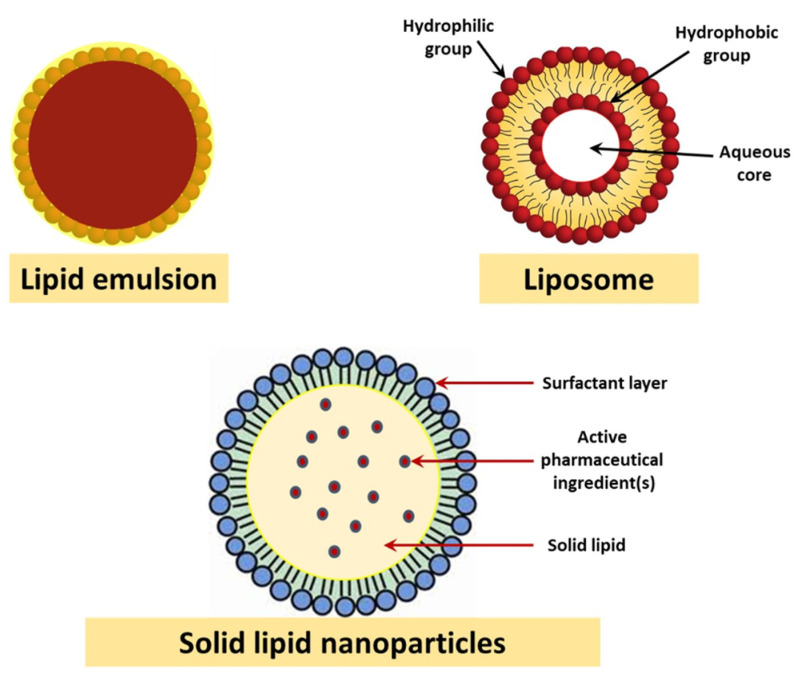
Different types of lipid nanoparticles used in the treatment of AD.

Figure 3 shows different types of lipid nanoparticles used in the treatment of AD. Touching leads to muscle cramps (thin-film hydration technique). To manufacture a lower layer formula, the Champagne is dissolved in an organic solvent. A thin layer of oil is left behind containing a solid surfactant and cholesterol. The water bath keeps the sheet swollen. The response kills drugs. Small particles are characterized by their size, shape, and release kinetics. The medium was 100.7 nm with a 0.232 index. The Zeta potential was measured and found to be −19 mV. The nanoparticles are examined using microscopic transmission electrons and smooth nanoparticles. Trap efficiencies ranged from 83.5% to 86.5% in formulations with improved bioavailability. Hence, they loaded the trap. The continuous release F2 formulation could deliver 60% in 240 min [48].

Nanoparticle delivery of drugs could prevent symptoms of Alzheimer’s. Studies in vitro show that Aβ_25—35_ may be prevented by synthetic α-bisabolol nanoparticles. Neuronal lines (donepezil-treated) have been shown to be more reactive and to have less potential for mitochondrial membrane and other benefits associated with mitochondria. ABS exposure appears to contribute to the protection of the brain [49]. An anti-medication Parkinson’s has been developed to transfer dipetin to the nanoscale to help make it easier for treatment for Alzheimer’s disease. The developer created a substance that was designed using rhodamine B-labeled solid lipid. Furthermore, particle size, dispersion index, zeta potential, infrared Fourier-transform spectroscopy, thermal analysis, and stability were to be measured to determine the samples. Metabolic and impedance methods have been tested for cytotoxicity and cellular conductivity of nanoparticles [50]. A human-made version of dextran-cholic acid (D × C) has been produced. Make a relatively simple machine to simplify the process of making nanoparticles. A gel material of a nanoparticle. High levels of the antimicrobial agent are used to cover the nanoparticles. These studies suggest that nanoparticles of maleic acid will penetrate the brain to give a new, more efficient protein product [51].

To ensure the safety and delivery of promising nanocarrier applications in long-term diseases, a sequence of NP, polymers and lipid-related diseases has been screened. Animals with intraperitoneal NPs were given in various tissues of their selected nanoparticles after 24 extremely concentrated doses. There were no improvements in toxicity, weight, or clinical signs. Repeated exposure in different clinical conditions of the NPs under review to the more promising chronic conditions NPs did not seem to be harmful [52]. The water maze test shows that spatial memory is restored. Treated rats are tested for cell absorption and bioaccumulation in their brain with computed tomography (CT) and histopathological examination. The fluorescent intensity transfersomes are 81.29% ± 2.64% higher than nanoemulsions. There were higher intensities. In relation to the formulation of nanoemulsions and pure drugs, the conduct acquisition of transfers and spatial memory in amnesic rats has increased significantly. CT refers to all brain-care rats’ accumulation of gold nanoparticles (GNPs), and higher GNP accumulation [53] was observed in rats that receive transfersome formulas.

The neuroprotective effect of green synthetic iron oxide nanoparticles was assessed using an aqueous extract from the entire *Convolvulus pluricaulis* (CPIO) plant in the amnesia-induced scopolamine model. CPIOs were evaluated orally for neuroprotective effects in scopolamine-induced amnesia mice at doses of 100 mg/kg, 200 mg/kg, and 400 mg/kg. Neurobehavioral studies have been conducted to test for learning and memory in mice using elevated plus labyrinth, Morris normal water labyrinth, and scopolamine-induced amnesia mice. CPIO 400 mg/kg demonstrated a significant improvement in the learning and memory of normal amnesia mice and scopolamine-induced mice in exteroceptive models.

Significant differences in lipid peroxidation, catalase and acetylcholinesterase have been established between 400 mg/kg of CPIO-treated amnesiac animals compared to untreated and scopolamine-treated group animals. The highest dose of CPIO had significant neuroprotective effects in normal and scopolamine-induced amnesia mice [54]. PLGA-soya lecithin-Tween−80 nanoparticles for AD have been developed. The response surface methodology (RSM) formulation was optimized using 32 factorial designs. Lipid polymer hybrid nanoparticles (LPHNPs) have been developed, consisting for the first time of a polymeric core and a phospholipid shell intertwined with PEG-based surfactants (SAA). Compared with the gold standard Tween−80, the objective of D-α-Tocopherol polyethylene glycol 1000 succinate (TPGS) or Solutol HS 15 is to improve brain transmission and prevent opsonization. Loaded with flavonoid rutin (RU) extracted from Calendula officinalis L using a modified single-phase nanoprecipitation technique.

Blossoms have been successfully prepared for LPHNPs and have recently been shown to be a promising anti-malarial product. Alzheimer’s effect of critical process parameter (CPP) and gold standard (Tween−80) critical quality attribute (CQA) concentration; trapping, size, and size distribution, statistically tested and optimized using the desired feature through experimental design. The optimized CPP was retained even though Tween-80 had been replaced by other PEG-based surfactants (SAA). A spherical shape was found in both hybrid particles with transparent lipid shells. The biocompatibility of the prepared NPs has been confirmed by the hemolysis test. Post-intravenous rat pharmacokinetic assessment showed significantly higher RU bioavailability for NPs compared to drug solution [55].

Biocompatible lipid membrane nanoparticles are extracellular vesicles; (EVs). These vesicles are secreted from different cells, such as mesenchymal stem cells (MSCs), and may pass through biological barriers to the transmission of information, such as signals, or may be used as carriers of different proteins, such as neprilysin, for example, (NEP). NEP is an active enzyme in the brain clearance of abnormally aggregated beta-amyloid sheets. EVs have been used in Alzheimer’s disease to help NEP recover from memory. The MSC of the bone marrow of the femur of the rat is isolated. The potency of differentiation and the precise flow-cytometry markers distinguished the assessment of the stemness of the developed cells. EVs were extracted from supernatant MSCs by ultracentrifugation and analyzed by electron microscopy (SEM), dynamic light dispersion (DLS) and Western blots. EVs were loaded with NEP through a freeze–thaw cycle and administered intranasally in the AD rat model for 14 days. EV-charged NEP induced decreases in IL-1beta and in BAX but increases in BCL2 expression in the brain of rats [56]. Antioxidant therapeutic effects of ellagic acid (EA) and EA-charged nanoparticles have been tested in the aluminum chloride-induced AD rat model (EA-NP). The EA load of nanoparticles was 0.84/1 *w/w*.

In vitro release of EA kinetics from EA NP to fetal bovine serum showed 60 percent release in the first 1–5 h followed by a sustained release at 60–70 percent over 6–24 h. Six groups were established; group 1 acted as a control, group 2 received EA, group 3 received EA−NP, group 4 administered 4 weeks of AlCl3 (50 mg/kg) to AD rats, group 5 (AD + EA) and group 6 (AD + EA−NP) administered 2 weeks of EA and EA−NP following discontinuation of AlCl3, respectively. Neurotoxicity in the brain of rats was investigated by measurements of catalase, glutathione, and full antioxidant activity and lipid peroxidation biomarkers in the brain [57]. Exosomes are lipid vesicles found on a nanometer scale in liquid biopsies and used as biomarkers for a range of diseases, including cancer, Alzheimer’s, and central nervous system diseases. Purification and subsequent sizing and surface characterization include the secrets of exosome-based diagnostics. Sample purification is time-consuming and potentially destructive, and there is no single calculation of the size and zeta potential for any current system. Exosomes were concentrated out of a dilute solution and measured their size and zeta potential in a one-step salt gradient measurement in the capillary tube.

The salt gradient causes the opposite direction of transport of particles and fluids that trap particles. The concentration of the particle increases by more than two orders of magnitude within minutes. Both their size and surface load are returned to match a single or a group of space distribution exosomes. To design the capillary, the capillary uses a low-cost system of polymers. Exosomes are used as disease biomarkers, but their characterization in biologic samples is difficult [58]. Cannabinoids have been shown to be effective in the treatment of various diseases, such as Alzheimer’s disease and multiple sclerosis. These advantages are hampered by their poor aqueous solubility, which decreases bioavailability. Lipid nanoparticles provide an effective alternative to improve the pharmacokinetics and biodistribution profiles of drug payloads. The size and efficacy of tissue penetration and liposome circulation are related to key considerations for these systems [59].

### 3.1. Role of Curcumin in Overcoming Aβ

AmyloLipid nanovesicles (ALNs) are the latest lipid-modified starch complex nanoparticles formed and presented as curcumin nanovesicles to attack the CNS through the intranasal direction. Curcumin has been shown to be a promising active agent with a range of pharmacological activities, including the ability to treat brain tumors, traumatic brain injury and CNS disorders, such as Alzheimer’s disease, as it can prevent the accumulation of amyloid-β-protein (Aβ) and inflammation caused by Aβ. Although curcumin has enormous potential for CNS disorders as a therapeutic agent because of its low bioavailability and rapid total body clearance, it reduces the potential for therapeutic levels to enter the brain. Mean brain concentrations of 141.5 ± 55.9 ng/g and 11.9 ± 12.0 ng/mL plasma concentrations were observed one hour after intranasal 160 μg/kg curcumin [60] administration using optimized precursor-produced ALN-charged curcumin.

Nanotechnology for curcumin and meloxicam co-nanoencapsulation in the treatment of neurodegenerative diseases has been proposed as a method. HPLC−DAD has developed and validated an analytical method for the simultaneous quantification of curcumin and meloxicam. Drug content, encapsulation efficacy, photostability and drug delivery were described as meloxican and curcumin plus meloxican lipid-core nanocapsules (LNCs). Toxicity levels for nanoparticles have been calculated in vivo (mice). Column C 18-RP was used when the guard column was filled with the same material as the fixed column. Acetonitrile: methanol: water: triethylamine (52:5:43:0.3 *v/v*/*v/v*) at 1 mL min^−1^ stream was used as a mobile phase. 424 nm (curcumin) and 365 nm (meloxicam) have been detected [61].

The nano-based therapies have been engineered to cross the blood–brain barrier and control it since they are smaller [62]. Curcumin is an effective natural anti-myeloid, anti-inflammatory, and antiapoptotic agent for many neurodegenerative conditions. The amyloid can alleviate stress, prevent neuronal damage, and restore normal AD functions for cognitive and sensory motors. Curcumin is fluorescent and ideally an incorrect Aß and can cause theranosis. Curcumin is, therefore, limited to low water solubility, lower biological abundance, and biological BBB failure [63]. Disease Mechanical, naive curcumin prevents the development of Aβ plates, attenuates and improves tau hyperphosphorylation clearance, binds copper, decreases cholesterol, changes micrographic activities, inhibits ac-acetylcholinesterase, promotes insulin signaling pathways, and is a highly effective antioxidant agent [64].

### 3.2. Role of Solid Lipids Nanoparticles (SLNs) in Treating AD

Niacinamide-loaded nanoparticles of phosphatidylserine/polysorbate 80 were synthesized, and phosphatidylserine (PS) or phosphatidic acid (PA) was applied. Careful considerations were given to the cytotoxicity, biodistribution, and speed of up taking of particles. We found that the optimum sizes of our molecules had values of 112 ± 1.6 nm, 124 ± 0.8 nm, and 137 ± 1.05 nm. S80-functionalized SLNs were highly toxic when applied in the SH-SY5Y cell line treatment. There is much recent research on the functions of hemoglobin., e.g., it is known that the PS-modified SLNs can prevent tau aggregation in the brain of Parkinson’s disease [65]. Cellular functionalized SLNs are absorbed more easily than SLNs functionalized with the corresponding antibody. A recently upgraded mouse monoclonal transferrin receptor antibody (OX-26) has enhanced transportability [66]. This drug is designed to treat or cure Alzheimer’s disease.

The levels of acetylcholinesterase (AChE) activities in the brains of animals that were administered with aniracetam were changed. Chrysin (CN)-loaded SLNs (CN-SLNs) could be used as a potential therapeutic and brain targeting strategy. It has been known that Aβ25-35 can cause memory decline. They can encapsulate the active ingredient [67]. Tarenflurbil (TFB) disclosed a crucial and news-making decision that was badly needed by the public. TFB was combined with polystyrene-PLA nanoparticles (TFB-SLNs). To use transdermal substances, 200 nanometers is recommended as it passes the nasal sensory nerves, and they have a diameter of 250 nanometers. TFB-NPs and TFB-SLNs have different efficiency levels of 64.11%. TFB NPs and SLNs have much better slow drug release properties than TFB pure drugs. TFB-SLNs and TFB solution had a more effective result than the nanoparticles. The effectiveness of the medicine is measured by the rate of absorption into the bloodstream [68]. This was how surfactants were used to separate the fats and water. The effectiveness of erythropoietin (EPO) combined with SLN in the prevention of Alzheimer’s disease was evaluated. EPO-SLN has proven effective in recovering memory. There is a beneficial impact on oxygen and energy metabolism in the brain because of EPO-SLN [69].

Swiss albino mice are used. The pharmacokinetics and pharmacodynamics of the SLN formulations have been performed. The bacoside-rich extract (BRE)-charged SLN was found to be much more potent than the BRE. Pre-made SLNs are better at maintaining drug release in mice for a prolonged period. Slices can be used to treat Alzheimer’s disease by deep brain stimulation through BBBs [70]. Due to its efficacy, usability, and flexible characteristics, its intellectual property potential is particularly valuable for pharmaceutical developers. Lipid nanoparticles are essential. All configurations contain nanoparticles with SLNs. Many lipid, thick, solid, and permeable liposomes were used for enhanced oil-in-water emulsions SLN [71].

Medicines have been utilized at the nanoscale to make medications easier for treating Alzheimer’s diseases. The developer created a substance that was decorated with Rhodamine B labels. The parameters investigated included particle size, dispersibility, zeta potential, infrared fast Fourier-transform (FFT) spectra, thermal analysis, and stability. Rivastigmine is a treatment for Alzheimer’s and works as an irreversible inhibitor of acetylcholinesterase using rivastigmine hydrogen tartrate-loaded tocopherol succinate-based solid lipid nanoparticles (RHT-SLNs). After conducting initial tests to establish the optimum particle size, the measurements were optimized further and confirmed [72].

Formulation of SLN as an alternative to oral and parenteral administration of water-soluble drugs called rivastigmine tartrate (RT) with Precirol ATO 5 and 80. The SLN was prepared using the hot, high-pressure homogenization method. The RT-loaded SLN has been secured for up to one month of storage. The zeta potential value of the SLN was found to be −10 mV, with an average particle size distribution of 214 nm in the polydispersity index range of 0.3–0.6. The Efficiency of encapsulation was found to be 59.23 percent. 160 ± 1.05-fold, 0.26 ± 0.05 mm, 98.63 ± 0.16 percent, respectively, were found to be RT-loaded transdermal patch folding endurance, thickness, drug–quality consistency. 4.2 ± 0.26 percent, 3.9 ± 0.31 percent, 9.23 ± 0.93 percent were found to be percent moisture content, percent moisture loss, and percent elongation loss. Ex vivo skin permeation and in vitro drug release kinetics showed 96.90 ± 0.69 permeation and 95.70 ± 0.87 percent drug release, respectively. The transdermal patch loaded with RT-SLN was prepared using Eudragit RS100, Eudragit RL100 and PEG 400 as plasticizers. The formulation has been optimized by a total of 32 Factory Design 10 experts. Full drug release was observed after 24 h in the ex vivo analysis [73].

Macrophage (MA) membrane-coated solid lipid nanoparticles (SLNs) have been developed by adding the rabies glycoprotein virus (RVG29) and triphenylphosphine cation (TPP) molecules for functional antioxidant delivery to neuronal mitochondria to the surface of the MA membrane (RVG/TPP-MASLNs). MA membranes have camouflaged SLNs by inheriting the immunologic properties of macrophages from the elimination of RES-rich organs. Following the RVG29 surface decoration, the ability to cross the BBB and selective targeting of the neurons revealed the special properties of the drug delivery system (DDS). The TPP also guided the DDS to mitochondria powered by electrical charging as the neurons entered the CNS. Genistein (GS)-encapsulated DDS (RVG/TPP-MASLNs-GS) has been shown to have the most beneficial impact on the relief of AD symptoms in vitro and in vivo through the combination of MA membranes RVG29 and TPP [74].

### 3.3. Merging Nanostructured Lipid Carriers (NLCs) and SLNs for Treating AD

Nanoparticles have been tested with metabolic and impedance methods for cytotoxicity and cellular conductivity. By targeting apolipoprotein E (APOE) protein, the level of lipid nanoparticles increases in the brain of non-human primates [75]. There are many remarkable applications of SLNs and nanostructured lipid carriers (NLCs). SLNs and NLCs are medicines often used to treat all kinds of diseases, such as for the treatment of cardiovascular and cerebrovascular diseases [76]. The potential of lipid nanocarriers, such as liposomes SLNs, NLCs, microemulsions and nanoemulsions, to enhance brain transport has been demonstrated, making it easier to enter the central nervous system (CNS) and allowing medicines that might benefit from the treatment of neurological disorders. Given the socioeconomic implications of these circumstances and the advent of nanotechnology, which ultimately contributes to the more effective and superior management of nanotechnology therapists, it is important to keep up to date with the latest understanding of these issues. BBB and pathophysiology have been created for major neurodegenerative disorders [77].

## 4. Inorganic Materials for Overcoming Aβ

Chemical compounds that do not contain carbon are referred to as inorganic materials (C). Glass, ceramics, and metals are examples of non-living materials obtained from non-living sources, such as rocks or minerals. Gold, silver, selenium, and iron are examples of inorganic nanomaterials that may be used to treat Alzheimer’s disease. The subsections that follow go over this position in greater depth.

### 4.1. Gold Nanoparticles Effects on Amyloid Beta Peptide

To classify Aβ, a gold electrode was updated with self-assembled mercaptopropionic acid (MPA), electrodeposited gold nanoparticles (AuNPs) and monoclonal antibody mAb DE2B4; all related experimental variables were optimized. Antibodies have been functionalized by chemical modification (thiolation) to allow the immobilization of antibodies with proper orientation on the surface of AuNPs, to enable the direct detection of Aββββ (1–42). Membrane Surface-Enhanced Raman spectroscopy observed the activity of Aβ on phospholipid membranes (MSERS). Phospholipid (PL) membranes consisting of 9:1 DMPC and DMPS molar ratios have shaped gold nanoparticles with a diameter of 100 nm (Au@PL). With an increase of approximately 40, Aβ increased the Raman Intensity Enhancement of Au@PL, and the H-bonding network was disrupted in the presence of Sodium chloride (NaCl), which surrounded Au@PL and separated Au@PL from each other. After mixing, the H-bonding network was disrupted when Aβ was applied to Au@PL. Aβ attracted the adjacent Au@PL as the reaction reached equilibrium and induced an Au@PL aggregation that blocked Aβ’s vulnerable aggregation to prevent further fibrillation. Based on our system, the behavior of the Aβ on the lipid membrane surface can be observed directly through enhanced Raman signals [78].

To characterize the biosensor construction, scanning electron microscopy, square-wave voltammetry, and electrochemical impedance spectroscopy were used. Aβ (1–42) was accurately identified using the proposed 10–1000 pg mL^−1^ linear immunosensor, with a maximum detection and quantification of 5.2 pg mL^−1^ and 17.4 pg mL^−1^, respectively; recovery values ranged from 90.3 to 93.6 percent for the measured spiking amounts. The immunosensor enables rapid, effective, precise, reproducible, and highly sensitive low-cost detection of Aβ (14.6% mL pg^−1^ reduction) and opens opportunities for ex vivo and in vivo diagnostic studies [79]. A hallmark of early-phase AD detection is the human plasma ratio of 40-and 42-residue amyloid β peptides (i.e., Aβ40 and Aβ42).

However, consideration may be given to important clinical applications for a non-antibody-based approach to simultaneous detection of Aβ40 and Aβ42. For visual detection of Aβ42 and Aβ40, the ‘nanoparticle-based colorimetric sensor array’ was constructed using label-free gold and silver nanoparticles. Different aggregation behaviors of nanoparticles have led to a variety of spectral and color changes through their conjugation with Aβ42 and Aβ40, followed by coordination between Aβ42 and Aβ40 and Cu (ii). A pattern recognition supervised approach, a linear discriminant analysis, and a quantitative difference between spectral changes (LDA). The proposed sensor array was able to distinguish at various concentrations between Aβ42, Aβ40 and HSA (50 nmol L^−1^ to 500 nmol L^−1^) and their mixtures. In human plasma samples, the sensor array had the capacity to detect structurally bound Aβ peptides [80]. For the detection of the apoE 4 gene, which is important for the early diagnosis of Alzheimer’s disease, a sensitive method has been developed. It is based on signal amplification using gold nanoparticles coated with streptavidin-modified ferrocene (Fc).

The additional apoE 4 gene catches the immobilized oligonucleotide probe. The simple identification of the GCGC sequences that are hydrolyzed by the HhaI restriction enzyme is followed by this. Cleavage only occurs in the complementary apoE 4 duplexes but prohibits uneven enzymatic cleavage. The apoE 4 series can be distinguished from the other apoE sequences. The limit of detection is as low as 0.1 pM of the ApoE 4 gene that benefits from the amplified signal and HhaI recognition of Fc-capped nanoparticle/streptavidin. Four blood-extracted genomic deoxyribonucleic acids (DNA) samples examined the existence of the apoE 4 gene. Viable proof of principle [81] will be given in the case of an enzyme-assisted electrochemical assay for the apoE 4 gene in genomic DNA by the method presented herein. Recent research has studied how the formation of Aβ aggregates affects the surface of gold nanoparticles to gain a thorough understanding of the mechanism of in vivo amyloid aggregation (AuNPs).

**Figure 4:**
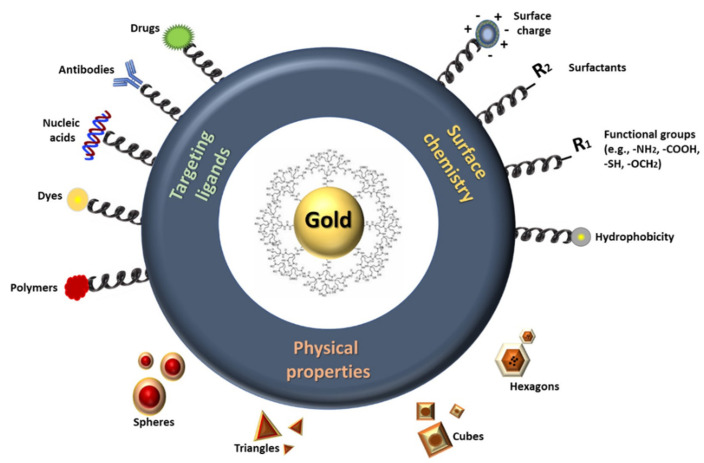
The structure of gold nanoparticles used in the treatment of AD.

Figure 4 shows the structure of gold nanoparticles used in the treatment of AD. To accelerate the formation of Aβ aggregates, AuNPs catalytically provide nucleation sites. In addition, AuNPs have great potential as a sensing tool owing to their optical properties. AuNP-based colorimetry is highlighted as a simple and creative method for monitoring the effectiveness of anti-Aβ reagents [82] using this dual purpose. The resulting crowns can produce several disease-specific proteins when nanoparticles (NPs) are exposed to these plasmas. AD and multiple sclerosis, utilizing gold NPs with different surface properties and coronal structure, have been developed to prevent and diagnose two neurodegenerative diseases. Separate protein compositions, including certain special proteins known as AD and MS biomarkers, have been used using several methods, including Ultraviolet (UV)-visible spectra, colorimetric response tests, and liquid chromatography-tandem mass spectrometry collected from various human serums. The promising capabilities of the technology to clearly recognize and differentiate between AD and multiple sclerosis (MS) are demonstrated by colorimetric responses, analyzed by chemometrics and statistical methods. The colorimetric technology developed could enable rapid, cheap, and rapid detection and discrimination of neurodegenerative diseases [83].

AD biomarkers suggest gold nanoparticle (AuNP) tags monitoring their electrocatalytic effect on hydrogen evolution (HER). The novel properties of porous magnetic microspheres (PMMs) are being exploited for the first time in terms of high functionality and high usable active area for the enhanced catalytic activity of the electrocatalytic tags of the captured AuNPs. The thorough characterization of the transmission of the high-angle, annular, dark field mode (STEM-HAADF) electron microscope shows that PMMs have improved their ability to collect a greater quantity of analyte and thus an electrocatalytic mark compared to microspheres that are commercially available [84]. Stabilized gold nanoparticles of 3.3 nm L-and D-glutathione were engineered and prepared. Without apparent toxicity, both chiral nanoparticles will inhibit Aβ42 aggregation and cross-BBB following intravenous administration.

Compared to its enantiomer L3.3, D3.3 has a higher affinity for Aβ42 and higher brain biodistribution, leading to increased Aβ42 fibrillation inhibition and better recovery of behavioral impairment in AD model mice [85]. In the rat animal model AD following intrahippocampal (IH) and intraperitoneal (IP) injections of the NP model, the production and retention of spatial learning and memory was studied (AuNPs). As demonstrated by reduced time (Aβ: 39.60 ± 3.23 s vs. Aβ + AuNPs: 25.78 ± 2.80 s) and distance (Aβ: 917.98 ± 50.81 cm vs. Aβ + AuNPs: 589.09 ± 65.96 cm) of secret platform discovery over training days and increased time spent on target quadrants (Aβ: 19.40 ± 0.98 s vs. Aβ + AuNPs: 29.366 ± 6.00), these particles could stimulate the acquisition and retention of spatial learning and memory in A-treated rats the unusual coordination of Cu2 + -Aβ-hemin, which facilitated the enrichment of microelectrode Aβ monomers, resulted in the assembly of Cu2 + -PEI/AuNPs-hemin nanoprobes on the microelectrode interface into network aggregates. The deposition of silver nanoparticles used in electrochemical stripping of Aβ monomers is facilitated by the AuNP aggregate.

Furthermore, increased selectivity against Aβ monomers [86] was observed. Using an in silicon deep neural network approach to detecting possible Aβ-42 inhibitors, a new screening technique was attempted. The library of PubChem compounds was screened, and wgx-50 was discovered. It was a potential Aβ-42 inhibitor. Compared to wgx-50 alone, the synergistic effects of the wgx-50-gold nanoparticles (AuNPs) complex induced substantial Aβ-42 inhibition. Molecular docking research, approach to system biology and simulation of the time course have verified that potential AD therapy applications will have complex synergistic effects of wgx-50-AuNP [87]. For the effective identification of Alzheimer’s disease biomarkers in human plasma using gold nanotubes with a chaotropic agent, a nanoplasmonic biosensor has been proposed. The localized surface plasmon resonance (LSPR), which is extremely sensitive to the point that the refractive index around gold nanoparticles reacts to insignificant changes, is the basis of this nanoplasmonic biosensor. Using guanidine hydrochloride as a chaotropic agent, blood-based AD diagnostic barriers could be overcome.

This agent disrupts the network of water molecules and weakens the interaction of proteins that are hydrophobic, and dramatically improves protein detection performance. By reducing the overlap between age-matched protein levels and AD patients’ plasma levels, this device can reliably diagnose AD patients. This instrument may also use a standardized blood tau protein biomarker associated with Alzheimer’s disease to analyze mild cognitive impairment [88]. For the detection of AD core biomarkers on a single platform via distinct localized surface plasmon resonance (LSPR) depending on the shape of gold nanoparticles, a highly selective biosensor called a shape-code biosensor has been proposed. This plasmonic sensor consists solely of gold nanoparticles and antibodies but does not require additional methods for accurate multi-sample separation and recognition. The detection limit of 34.9 fM for 1–40 amyloid-beta (Aβ), 26 fM for 1–42 aβ, 23.6 fM for Proteins corresponding to variations in Rayleigh peak dispersion of ~1, ~2.23 and ~3.12 nm in the plasma form-code system have been measured for each blood mimicking biomarker [89] under physiological conditions.

To boost the sensitivity of the assay, the nanocomposite surface (rGO-AuNP) has been changed to serve as a covalent anchor with 11-mercaptoundecanoic acid (11-MUA). Using EIS data, the surface coverage value and the pinhole ratio were computed. Furthermore, measured is Kramers-Kronig data, which helps interpret instrument errors. In accordance with the single frequency impedance, the tau-441 anti-tau immune response was controlled (SFI). Using scanning electron microscopy (SEM), atomic force microscopy (AFM) and Fourier-transform infrared spectroscopy, surface morphology changes were assessed (FTIR). The engineered tau-441 analytic immunosensor target demonstrated a linear response within a concentration range of 1–500 pg/mL and a detection limit of 0.091 pg/mL. The study’s encouraging argument is that in this neurobiosensor, both serum fluid and cerebrospinal fluid (CSF) samples with recovery rates ranging from 96 to 108 percent can catch tau-441 target proteins [90]. For the S100ß protein, a biomarker of Alzheimer’s disease found in brain astrocytes, a sandwich-type photoelectrochemical immunoassay was identified. The S100ß (anti-S100ß) antibody was labeled with quantum dots of CdS and treated as a secondary antibody.

FTIR, ultraviolet and fluorescence spectroscopy is used to classify the called antibody. From the indium-tin oxide (ITO) electrode, a nanocomposite of reduced graphene oxide and gold nanoparticles has been updated. A film was then applied to the electrode surface containing functional isocyanate groups (−N = C = O). To covalently bind them to the surface, the NCO group communicates with the amino groups of the labeled antibody. The S100β was connected by the primary immobilized antibody to the rGO-Au/ITO electrode and then sandwiched to the labeled secondary antibody. To validate subtle changes in the electrochemical properties of the electrode surface [91], cyclic voltammetry and electrochemical impedance spectroscopy have been used.

Treatment with AuNP according to the AD model, okadaic acid (OA) was assessed. Male Wistar rats were injected with OA (100 μg) intracerebroventricularly and treated 24 h later with 20 nm AuNP (2.5 mg/kg) for 21 days every 48 h. Sham, AuNP, OA, and OA + ANP were divided into the following groups (n = 12/group). Although AuNP is still common in the cortex and hippocampus, OA increases the phosphorylation of tau. OA has influenced spatial memory, and this deficit has been avoided by AuNP therapy. OA has been decreased by neurotrophic factors (BDNF and NGF-β) in the cortex and hippocampus. In the hippocampus and cortex, the OA and OA + AuNP groups had increased interleukin (IL)-1 β, and the AuNP group had increased IL-1 β in the hippocampus. In the two classes, the cortex and hippocampus levels of S100 were increased by OA. IL-4 increased in the OA + AuNP species. AuNPs in brain structures caused by OAA has withstood oxidative stress (sulfhydryl and nitrite levels).

Normal brain mitochondrial function has been maintained by OA modulated ATP synthase activity and AuNP. OA reduced brain antioxidant potential, and AuNP (SOD, catalase and GSH activity) restored antioxidant status [92]. Based on its fundamental significance in the field of life and nature, nanoscale chirality has drawn substantial interest from different fields of research. For the treatment of Alzheimer’s disease, D-/L-Pe-Au nanoparticles, chiral penicillamine-modified gold NPs, have been developed and made. Using rat PC12 (pheochromocytoma) cells, a real-time cell analysis assay was performed to investigate the possible cytotoxicity of d-/l-Pe-Au nanoparticles [93]. Sporadic AD models were used to test the therapeutic effect of gold nanoparticles in rats with intracerebroventricular streptozotocin (i.c.v.-STZ) injection (GNPs). The null hypothesis that there would be no difference between the STZ + GNPs group and the STZ group in the markers examined was confirmed. For the prevention of GNP treatment, STZ-induced impairment of mitochondrial ATP output, neuroinflammation and oxidative stress have all been shown.

Furthermore, while the STZ induced both spatial and recognition memory deficits, the GNP [94] prevented this effect. Interdigitated microelectrodes (IMEs) were produced for the blood-based detection of Aβ using gold nanoparticle (AuNP) sandwich assays as an impedance biosensor. It supported linear, logarithmic sensitivity and approximately 2.87 and 74.84 percent improved detection limits. The mouse plasma sample was prepared from the blood mouse groups of dual-mutated APP/PS1 transgenic (TG), and wild-type (WT) and the AD diagnostic capability was evaluated in the plasma samples by Aβ detection. It has been shown that, by helping to detect Aβ, the AuNP sandwich assay has effectively discriminated against the TG and WT mouse types. With high sensitivity and selectivity, Aβ was observed. This Aβ sensing device with AuNP sandwich assay will lead to major advances in human blood sample clinical diagnosis [95].

The manufacture of 11-mercapto-1-undecanesulfonate-coated gold nanoparticles (NPs) has been reported to effectively mark the edges of synthetic, recombinant, and native amyloid fibrils derived from different amyloidogenic proteins. For the evaluation of amyloid morphological polymorphism by cryogenic transmission electron microscopy (cryo-EM) [96], these NPs were effective methods. With HS-PEG-OMe and HS-PEG-COOH, GNRs have synthesized, modified, and functionalized the D1 peptide, which has the potential to be selectively bound to the amyloid beta-peptide. We coincubated amyloid beta-peptide aggregates with CRANAD-2 and GNR-PEG-D1 probes to detect in vitro amyloid beta-peptide detection and to recognize an increase in fluorescence signal intensity due to improved surface fluorescence. In transgenic mice with Alzheimer’s disease co-incubated with CRANAD-2 and GNR-PEG-D1, a surface fluorescence effect was observed in the brain. Increases in the fluorescence signal for the detection of aggregates that cannot be detected by the single use of CRANAD-2 have been identified. Gold nanoparticles have made it possible to enhance the in vitro and ex vivo detection of amyloid fluorescence aggregates [97].

Parkinson’s disease (PD) is the second most common neurodegenerative disorder after Alzheimer’s (PD). Reserpine administration to animals as a PD model was suggested based on the motor activity effects of this monoamine-depleting agent. When used at certain concentrations, gold nanoparticles (GNPs) are effective in the treatment of neurodegenerative disorders. Under behavioral and oxidative stress conditions, the effects of GNP administration were tested in an experimental PD model. The animals were divided into four classes (N = 6): sham; sham and GNP; reserpine; reserpine and GNP; C57BL/6 mice for 40 males (20–30 g). Three doses subcutaneously were administered at 48 h intervals with a reserpine concentration of 0.25 mg/kg [98]. Using light scatter absorption, fluorescence, TEM, CD spectroscopy and SDS-PAGE, the effect of gold nanoparticles made from hibiscus on the amyloid formation of alpha-lactalbumin was assessed. Due to the shape of molten globulin, lactalbumin was selected as a good sample for the amyloid formation analysis. The AuNPs inhibit the development of al-lactalbumin-reduced amyloid fibers. The consequence of this defensive effect is that nanoparticles’ adsorption of proteins to the surface improves and avoids structural changes. Nanoparticles prevented them by attaching them to the monomer from accessing and extending the amyloid fibrils in the middle [99].

The effect of pro-AuNP on hen egg-white lysozyme (HEWL) fibrillation was investigated using thioflavin T (ThT) and 8-anilino-1-naphthalenesulfonic acid (ANS) studies. The natural sigmoid character of protein aggregation was demonstrated by HEWL kinetics and adapted to the Boltzmann model. In the presence of bare gold nanoparticles, HEWL has shown comparable aggregation kinetics to HEWL alone (bAuNPs). HEWL fibrillation significantly decreased when coincubated with proline and pro-auNP, and two slightly different intermediate species with these two systems were formed [100]. Neurotransmitters synthesize dopamine (DA) and its synthetic precursors (L-phenylalanine (L-Phe) and L-tyrosine (L-Tyr)) and combine them with ultrasmall gold nanoparticles (USGNPs, ~4 nm in diameter). These functionalized USGNPs can effectively inhibit the Aβ fibrillation stage, mainly because of the spectroscopic growth points following the anchoring of functionalized USGNPs to short seeds, as well as the peptide solution folding mechanism revealed by the measurements of the spectroscopic transmission electron microscope (TEM) and circular dichroic (CD) (sequence of m sequences) E The A This is of vital importance for the development of novel AD drugs because oligomers are the primary source of Aβ toxicity [101]. Compared to in vitro Aβ1-42-injected mouse and AD models, neuroprotective effects were observed for anthocyanins and anthocyanin-charged poly (ethylene glycol)-gold nanoparticles (PEG-AuNPs) and poly (ethylene glycol)-gold nanoparticles (PEG-AuNPs). Anthocyanins alone or in combination with PEG-AuNPs (ANPEG-AuNPs) have been shown to inhibit in vivo and in vitro AD models of p-JNK/NF-egB/p-GSK3β pathways activated by Aβ1-42 to suppress neuroinflammatory and neuroapoptotic markers. Anthocyanins packed with PEG alone are stronger than anthocyanins [102]. It was suggested to use a colorimetric sandwich immunosensor based on dual antibody-adjusted gold nanoparticles for use in Aβ (1-42). Bare AuNP has been successfully coated with the N-terminal antibody Aβ (1–42) (N-Ab (1–42)-AuNP) and the C-terminal antibody Aβ (1-42) (C-Ab (1-42)-AuNP). Because of the particular binding of N-and C-terminal antibodies to Aβ (1-42), Aβ (1-42)-AuNP: N-Ab (1-42)-AuNP = 1:1), prepared AuNPs@C/N-Ab (1-42) (C-Ab (1-42)-AuNP: N-Ab (1-42)-AuNP = 1:1) and aggregated AuNPs@C/N-Ab (1-42) can be captured simultaneously, accompanied by a big color change from red to blue.

High linearity is observed between 7.5 nM and 350 nM with a detection limit of 2.3 nM using this colorimetric sandwich immunosensor system, which is equivalent to or better than the other detection methods reported for Aβ (1-42) [103]. In a well-established in vitro model setup, the effect of gold nanoparticle size, surface load, concentration, and morphology on the integrity of the blood–brain barrier was examined (BBB). The effect has been localized in hollow gold nanospheres and gold peptide-functionalized nanotubes selectively bound to amyloidogenic β-amyloid structures. In AD therapy in vitro, these AuNP conjugates have already been successfully tested for possible use as photothermal absorbers, but they may have a poor passage through the BBB due to their overall negative load [104]. Several biophysical techniques have been established as-synthesized nanoparticles, such as ultraviolet-visible (UV-vis) spectroscopy, transmission electron microscopy (TEM), X-ray diffraction (XRD), dynamic light scattering (DLS), zeta-potential measurement and Fourier-transform infrared spectroscopy (FTIR). Aggregation research has shown that a partial HEWL amyloidogenesis regulator is PVP. When conjugated to the surface of the gold nanoparticle, it leads to the optimal inhibition of amyloid formation.

PVP-conjugated gold nanoparticles have also shown important disaggregating effects on mature amyloids in addition to inhibition and can, therefore, be used as an effective therapeutic agent against inherited systemic amyloidosis [105]. A label-free, sensitive, and selective visual and fluorescent detection method for AβOs based on internal filter effects (IFE) for CdTe quantum dot fluorescence gold nanoparticles (AuNPs) has been written (QDs). AuNPs by IFE has substantially extinguished the fluorescence of CdTe QDs. Mediated aggregation and color shift in AuNP suspension, PrP (95–110), a cellular prion protein AβO-specific binding peptide, weakened AuNP’s IFE to CdTe QD fluorescence and restored fluorescence power. The absorption of PrP (95–110) on the AuNP surface was prevented by the unusual interaction between AβOs and PrP (95–110) in the presence of AβOs. Accumulation of AuNPs was stopped, and the fluorescence intensity of CdTe QDs was again extinguished. For the identification of AβOs, but not AβMs and AβFs, this label-free technique is distinctive. Detection limits for visual assays were found to be 0.5 nM and for fluorescent detection to be 0.2 nM [106].

To detect the formation of Aβ amyloid fibrils and oligomers, a gold nanoparticle (AuNP) study was performed. The surface plasmon resonance (SPR) absorption band amplitude of AuNPs is sensitive to the number of amyloids present in Aβ40. This helps to use the SPR test to categorize and semi-quantify Aβ40 amyloids and explain the kinetics of forming Aβ amyloids. AuNPs’ SPR band strength is susceptible to the existence of mutant oligomers of Aβ40 and Aβ40, which form more stable oligomers. After a change in the SPR band strength of AuNPs, the kinetics of stable oligomer formation of the mutant Aβ40 can also be regulated. Mechanistic studies of early self-assembly proteins and fibrillogenesis [107] can use this nanoparticle-based approach. Evidence has shown that brain pathology has been caused by functional AuNPs, depending on the size, dosage, and route of administration. These AuNPs activate blood–brain barrier permeability with protein tracers that cause brain edema production and neuron and glial cell injury (BBB). The degree and level of brain disease caused by AuNPs are inversely proportional to the size of the NPs. Interestingly, through co-administration of cerebrolysin, a controlled combination of multiple neurotrophic factors and active peptide fragments, AuNP brain pathology is reduced. This effect was even more pronounced when, using TiO2 nanowired delivery, cerebrolysin was administered, which was not previously indicated [108]. A sensitive and reliable microRNA test was submitted based on the colorimetric detection of gold nanoparticles and amplification of the hybridization chain reaction (HCR).

The need for enzymatic reactions, chemical changes, separation processes and specialized equipment is minimized by this technique. The detection process is obvious with bare eyes, and the maximum of detection for this technique is 0.25 nM, which is less than or at least closer to the previous colorimetric AuNP methods. The high sensitivity and specificity to differentiate between perfectly matched, inconsistent, and noncomplementary target microRNAs and a strong response in the analysis of actual blood plasma samples are the important characteristics of this approach [109]. Alzheimer’s disease (AD) biomarker (Aβ1-42) (Aβ1-42) (Aβ1-42) amyloid-beta (1-42) (Aβ1-42) (Aβ1-42) recognition sensors based on localized surface plasmon resonance (AD) (LSPR). The sensors included ligand-exchanged gold nanoparticles (Au NPs), which were stored using the Langmuir-Blodgett (LB) process in a polyethylene terephthalate substrate. Using streptavidin as a biotin conjugate to LB ligand-exchanged Au NP film, monoclonal antibodies (anti-Aβ1-42) were then immobilized.

The binding of biomarkers to antibodies immobilized to the LB film has been observed by measuring the absorption change in the plasma peak response. With contrasting effects of various sizes and film thicknesses, the sensor structure was designed for Au NP LB films. The integrated sensor was used to detect biomarkers at different concentrations in the buffer and dilute cerebrospinal fluid (CSF) solutions [110]. Table 1 introduces a comparison between different types of nanoparticles used in Alzheimer’s disease treatment.

### 4.2. Silver Effects on Amyloid Beta Peptide

The restorative capability of a fluid concentrate of Nepenthes khasiana leaf as a diminishing and balancing out specialist for AgNP combination has been researched in irregular Alzheimer’s illness model rodents created by intracerebroventricular infusion of streptozotocin (i.c.v.– STZ). The AgNPs were grouped utilizing a mix of spectroscopic and minuscule procedures as they were ready. Face-focused cubic (FCC) glasslike AgNPs were found utilizing an XRD design [111]. The arrangement of silver nanoparticles was affirmed utilizing a transmission electron magnifying instrument (TEM), UV-noticeable spectroscopy, Fourier-transform infrared (FTIR) spectroscopy, dynamic light dissipating (DLS), X-beam diffraction (XRD), and energy-dispersive X-beam diffraction (EDXRD) (EDX). These SNPs featured its promising potential as a plant-based enemy of Alzheimer’s prescription and against oxidative pressure regarding anticholinesterase and cell reinforcement action [112].

Silver nanoparticles (AgNPs, 50 g/mL), cerium oxide nanoparticles (CeO2NPs, 100 g/mL), and cadmium telluride quantum spots (CdTeQDs, 3 or 10 g/mL) were utilized to survey the limit of mouse BV-2 microglia to clear A plaques. Microglial cells help at the end of A plaques from the mind. The trypan blue test and propidium iodide restricting were utilized to decide cell suitability and cycle movement separately. Stream cytometry was utilized to quantify the assimilation of An and NPs. The two NPs examined affected microglia phagocytic action (AgNPs and CeO2NPs) and additionally reasonability (AgNPs and CdTeQDs), recommending that they could help delay the inception and movement of Alzheimer’s infection [113]. Protein fibrillar total amassing and affidavit in tissues have been ascribed to numerous neurodegenerative problems, including Alzheimer’s and Parkinson’s. Protein conglomeration is accepted to be forestalled by sub-atomic chaperones, which are proteins all by themselves. The impact of different groupings of green union silver nanoparticles (AgNPs) on-lactalbumin (-LA) conglomeration and s-casein chaperone activity in Pulicaria undulata L. was examined [114]. The specialists made another SERS-based sandwich immunoassay utilizing tannin-covered silver nanoparticles and attractive graphene oxide (Fe3O4@GOs). In the wake of utilizing this technique to recognize protein norms in cradle arrangement, the relapse condition was created. It was then scrutinized on Alzheimer’s sickness serum tests to perceive how effective it was. The SERS-based immunoassay set up effectively surveyed A1-42 and P-tau-181 in human serum examples, making it a promising strategy for early recognition of Alzheimer’s infection [115].

The analysts built up another sandwich-type biosensor for the electrochemical identification of-1 antitrypsin (AAT), an Alzheimer’s sickness biomarker. The sign enhancer was Snowcapped mountain AAT Abdominal muscle Ag NPs, and the detecting medium was 3, 4, 9, 10-perylene tetracarboxylic corrosive/carbon nanotubes (PTCA-CNTs) [116]. Another exploration utilized a triple cell co-culture model comprised of mouse cerebrum endothelial (bEnd.3) cells, mouse mind astrocytes (ALT), and mouse neuroblastoma neuro-2a (N2a) cells to see whether AgNPs disturb mind endothelial cells’ close to intersection proteins and modify neuronal cell proteomic digestion. AgNPs aggregated in ALT and N2a cells considering the interruption of close to intersection proteins claudin-5 and ZO-1 in bEnd.3 cells, as indicated by the outcomes. After AgNP openness, proteomic profiling of N2a cells uncovered 298 differentially communicated proteins connected to unsaturated fat digestion. In N2a cells, AgNP-actuated palmitic corrosive advancement was noticed, which may energize A creation [117].

### 4.3. Selenium Effects on Amyloid Beta Peptide

The organic activity and medication conveyance properties of curcumin nanoformulation in the Alzheimer’s infection therapeutics can be created by the adjusting surface of PLGA polymer and embodiment of selenium nanoparticles (Se NPs). The morphological design, size conveyances of nanospheres, synthetic associations between the polymer and nanoformulations of combined curcumin and Se NPs stacked PLGA nanospheres have been concentrated by utilizing the methods of scientific instruments [37]. The impact of selenium-chondroitin sulfate nanoparticles (CS@Se) has been learned on multi-target-coordinated treatment for the treatment of Alzheimer’s infection (promotion). CS@Se nanoparticles were effectively blended, and their restorative impacts were concentrated in vitro promotion models. CS@Se viably repressed amyloid-β (Aβ) accumulation and shielded SH-SY5Y cells from Aβ1-42-prompted cytotoxicity [118]. Two focusing on peptides (LPFFD and TGN) were formed from selenium nanoparticles (SeNPs).

It is tracked down that the focus proportion of LPFFD to TGN taken as 1:1 could frame the best double utilitarian SeNPs (L1T1-SeNPs) for restraining Aβ collection and the intersection of the BBB. L1T1-SeNPs can cross the BBB and have a solid liking toward Aβ species, and in this manner, they can productively stifle extracellular Aβ fibrillation by disturbing hydrophobic and electrostatic connections that are significant for Aβ40 nucleation [119]. Chiral penicillamine-covered selenium nanoparticles (l−/d−Pen@Se NPs) have been planned and synthesized that can go about as a novel class of chiral amyloid-β (Aβ) inhibitors. The d-Pen@Se NPs have exhibited higher restraint proficiency, just as improved comprehension and memory hindrances. We utilized rodent pheochromocytoma (PC12) cells to perform constant cell examination measures (RTCA) to test the likely cytotoxicity of l-/d-Pen@Se NPs. At some random time, the cell record diminishes as d−Pen@Se NPs fixation increments, exhibiting a focus subordinate cytotoxic impact on PC12 cells [120]. Sialic corrosive (SA)-changed selenium (Se) nanoparticles formed with an elective peptide-B6 peptide (B6-SA-SeNPs, an engineered selenoprotein simple) have been synthesized, which shows high porousness across the BBB and can possibly fill in as a novel nanomedicine for sickness alteration in Advertisement. Laser-examining confocal microscopy, stream cytometry investigation and inductively coupled plasma-nuclear discharge spectroscopy ICP-ES uncovered high cell take-up of B6-SA-SeNPs by cerebral endothelial cells (bEnd.3) [121]. Metals could incite Aβ collection by their redox action or restricting properties to amyloid β fibrils, prompting their gathering and testimony outside neurons. Consequently, metal chelation may have a recognized part to play in Advertisement counteraction and treatment.

The job of various selenium species, including selenium nanoparticles, in Aβ collection was concentrated on assessing their metal-chelating properties and their capacity both to repress metal-instigated Aβ 1–42 total fibrils and to disaggregate them once shaped. Progress biometals like Fe (II), Cu (II), and Zn (II) at 50 μM were chosen to build up the in vitro models. The DPPH test was utilized to decide the cancer prevention agent limit of the assessed selenium species [122]. The novel Aβ assimilation property of selenium nanoparticles has been joined with the normal cell reinforcement specialist Res to frame Res@SeNPs. In vitro organic assessment uncovered that adjustment of Res with SeNPs gives a synergistic impact on Cu2 + −initiated Aβ42 conglomeration, ROS age, and more critically, shields PC12 cells from Aβ42-Cu2 + buildings incited cell demise. It is accepted that SeNPs can improve the utilization of Res in Advertisement treatment as Res@SeNPs are more productive than Res in diminishing Aβ42 poisonousness in long haul use [123].

### 4.4. Iron Effects on Amyloid Beta Peptide

As new theranostic experts for the company, ultrasmall superparamagnetic iron oxide nanoparticles coupled with a phenothiazine-based near-infrared (NIR) fluorescent tone have been produced and inspected. They would be able to use in vivo NIR fluorescence and enchanting reverberating imaging of A plaques in the cerebrums of twofold transgenic mice to avoid A crowd, disaggregate preformed A fibrils, and exert a careful impact on the toxicity of human neuroblastoma cells induced by A1-42 [5]. W20/XD4-SPIONs are multifunctional nanoparticles made by grafting oligomer-univocal scFv immunizer W20 and class A scrounger receptor (SR-A) activator XD4 onto superparamagnetic iron oxide nanoparticles (SPIONs). Amazing light disseminating and transmission electron microscopy were used to investigate the SPIONs’ tenacity and size consistency. Immunocytochemistry and stream cytometry evaluations highlighted the importance of W20/XD4-SPIONs for detecting A oligomers (AOs) and progressing AOs phagocytosis. A co-culture model was used to create the blood–cerebrum hindrance vulnerability of W20/XD4-SPIONs.

The advancement of W20/XD4-SPIONs in vivo in progress mouse minds was observed using enticing resonation imaging (X-shaft) [124]. It is thought that quercetin-shaped superparamagnetic iron oxide nanoparticles (QT-SPIONs) have a neuroprotective effect on movement when compared to free quercetin and regulate threat avoidance, apoptotic, and application efficiency as well as miRNA-101. Male Wistar rodents were exposed to AlCl3, AlCl3 + QT, AlCl3 + SPION, and AlCl3 + QT−SPION for 42 days in this experiment. The abundance of solutions was assessed using social tests and qPCR. The power of mental deficiency was decelerated at both the middle and end of the treatment time span, according to the potential consequences of lead tests. The impact of QT-SPIONs on learning and memory requirements was enthusiastically received during the benchmarking process. The advancement of promotion achieved in rodents treated with AlCl3 was prompted by progress in explanation levels of use efficiency and a decrease in mir101, although these findings were reversed in the AlCl3 + QT−SPIONs pack [125]. Instead of monomers or fibrils, A oligomers (AOs) are thought to be the essential neurotoxic organisms. Restorative procedures that target AOs and advance A chance may have a surprising effect on business care. With A oligomer-unequivocal scFv safe reaction W20 and class A scrounger receptor activator XD4 (W20/XD4-SPIONs), a multifunctional superparamagnetic iron oxide nanoparticle has been shaped.

Aside from the consistent worth, W20/XD4-SPIONs maintained W20 and XD4’s anti-A properties by preventing A gathering, reducing AO-activated cytotoxicity, and inducing A phagocytosis in microglia. W20/XD4-SPIONs effectively covered mental requirements and eased progress mouse neuropathology when applied to application/PS1 mice [126]. 1,2-dioleoyl-sn glycerol-3-phosphoethanolamine (DSPE)-n-[poly (ethylene glycol) (PEG)] stacked with canine and superparamagnetic iron oxide (SPION) formed with two targets ligands to the outside of the nanoparticles, CRT and QSH, decreased as SPIO@DSPE-Stake/Mutt CRT/QSH. CRT unmistakably targets ligands at the blood–brain barrier (BBB), and QSH has a strong affinity for A1-42, the responsible group in Headway pathology. Amazing light scattering (DLS), transmission electron heightening position of intermingling, polarization submersion, and stream cytometer evaluation were among the in vitro cutoff points for nanoparticles [127].

## 5. Discussion

Nanoparticles play an important role in treating AD. Numerous reaction locations and good drug-loading charges are given by DGLs-PEG-RVG29-D-peptide/DNA NPs, which are referred to as D-peptide/DNA NPs. Codelivery of blood–brain barrier-crossing drugs was shown in vitro and in vivo to be effective when administered using brain-oriented ligand modifications. Drug-loaded PLGA nanoparticles enhance the spatial memory and recognition of transgenic AD mice while also playing an important role in transgenic AD mice. In vitro on SH-SY5Y cells, PLGA@QT NPs have low cytotoxicity. 50 × higher brain endothelium absorption than free drug and displayed a delayed in vitro release profile of PGZ were achieved by using PGZ-NPs carriers. NPs coated with poly (acrylic acid) liposomes that contain an anti-A mAb are stable in serum protein incubation and in a position to bind to in vitro monomers and fibrils. Immune nanoparticle uptake was substantially increased when administered to the body with peptide iA5 delivery in the absence of monoclonal antibody function.

As the negative T2 contrast agent, the calming rate of PiB-R2 MZF was found to be 169.93 mM^−1^S^−1^, high superparamagnetism. In two cell lines, PiB-MZF was found to be non-toxic. The nonpathogenic pathogenic (NP (α-M)) can increase the clearance of Aβ-124I-radiolabeled aggregated amyloid-β42 (Aβ1–42) in an LDLR-dependent manner, decreasing Aβ deposition and reducing the neuroinflammatory responses. This property of Eu/GMP ICP means that it has a self-adaptive function, which means it can alter the competitive coordination interaction of Cu2þ between the guest CDs and Ab monomer, according to their need. The Se-PLGA nanosphere drug delivery system was loaded with a Cur-loaded Se-PLGA nanosphere drug delivery system, which results in decreased amyloid-β load in the brain samples of AD mice and provides significant assistance in treating the model mice ‘memory deficit. Iron oxide nanoparticles that are synthesized in a biologically relevant manner have not been found to negatively impact neuroblastoma cell viability.

The administration of DBP-PLGA nanoparticles, through an intravenous injection, greatly reduced the amount of Aβ accumulation, the severity of neuroinflammation, neuronal death, and cognitive impairment in the 5XFAD mice. There are non-cytotoxic brain cell lines originating from MEM-PEG-PLGA. NPs that had been released into the free medication solution over a longer period were added into the body through a slower release profile and thus limited the in vivo drug control frequency. The spatial learning and memory capacities of APP/PS1 mice could be significantly enhanced with the use of PLGA-PEG-B6. In vitro bifunctional nanozyme activity was demonstrated with MoS2 QDs and TPP-MoS2 QDs, which are efficient in reducing spontaneous neuroinflammation. SLNs engineered to have the abilities of S80, PS, and PA proved more effective at ameliorating the cognitive disability of rats than the administration of nicotinamide on its own.

The therapeutic capacity of CN can be achieved with lower doses, while the bioavailability of oral CN can be increased by encapsulating CN in SLNs. Following the intranasal administration of polymers and lipid nanoparticles, TFB may be delivered directly to the brain’s olfactory system, where it would have a therapeutic effect. For the amyloid-beta study, it was found that NLC permeates the blood–brain barrier, while for NLC-transferrin studies, it was discovered that they have the capacity to inhibit fibril formation. Epo-SLN has the potential for increased anti-colon cell efficacy and better drug encapsulation. When the coating formula had a positive charge, the particles were mucoadhesive and remained in the nasal cavity for a longer period. Sixty years of age and elevated cholesterol were needed to solve the widespread rapid metabolism of rivastigmine.

**Figure 5:**
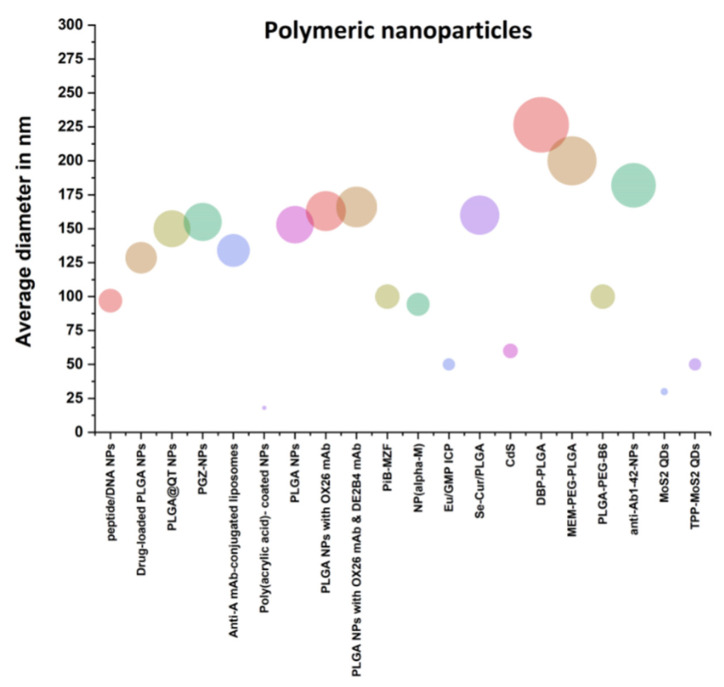
Different sizes of polymeric nanoparticles used in AD treatment.

**Figure 6:**
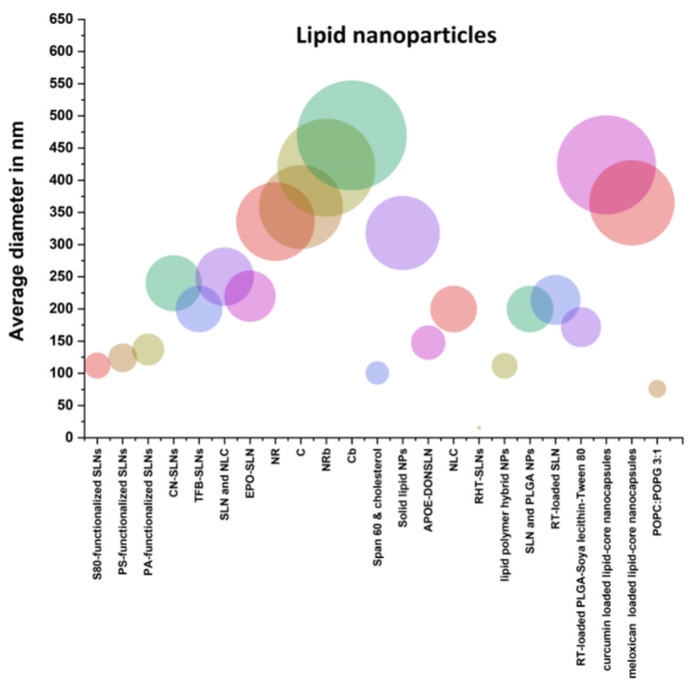
Different sizes of lipid nanoparticles used in AD treatment.

**Figure 7:**
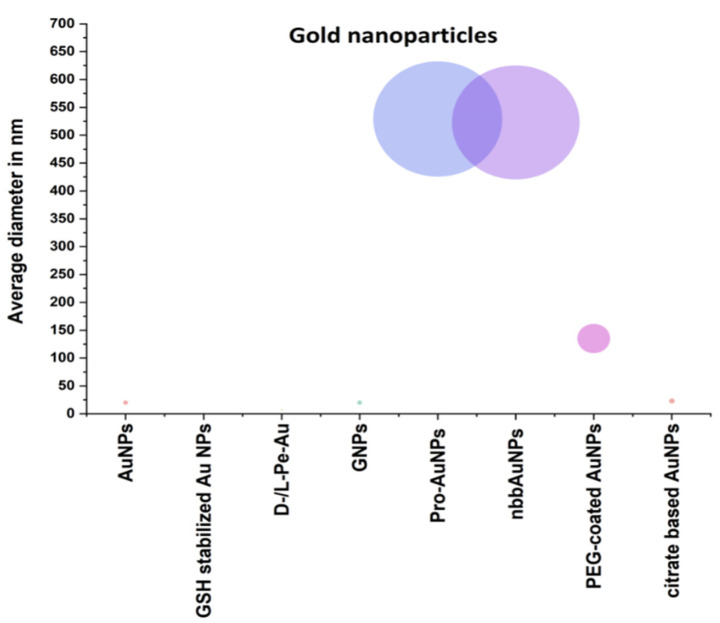
Different sizes of gold nanoparticles used in AD treatment.

To enter solid lipid nanoparticles, the ApoE molecule that is unique to BBB receptors can be exploited. The toxicity against the SH-SY5Y cell line is very mild. Different sizes of polymeric nanoparticles are shown in Figure 5. Moreover, different sizes of lipid nanoparticles are shown in Figure 6. Furthermore, different sizes of gold nanoparticles are shown in Figure 7. Quick, direct penetration of bio-inspired surface-modified NPs was observed when lipid polymer hybrid NPs were used. There were no side effects, increases in body weight or diseases observed. When the zeta potential of 10 mV was discovered, the polydispersity index was discovered in the range of 0.3 to 0.6. An open-label pilot study is being performed to see whether modafinil is an appropriate treatment for Alzheimer’s disease and to assess if it could act to administer long-term radiation therapy to the brain.

The catalytic activity of captured AuNPs electrocatalytic tags is enhanced by using high functionality and a highly active region. Unlike its GSH-stabilized L-and D-glutathione (GSH) counterparts, chiral L-and D-glutathione (GSH) Stabilized Au NPs do not exhibit toxicity to BBB when intravenously administered and are also capable of inhibiting Aβ42 aggregation. The cell index of PC12 cells decreased significantly by the addition of D−/L−Pe−Au, suggesting that cytotoxic effects on these cells are dose-dependent. GNP-treated mice: Therapeutic potential of GNPs, as measured in behavioral and oxidative stress parameters.

By diminishing inflammation and oxidative stress, the medical potential can reduce the progression of secondary neurodegenerative conditions and reserpine-induced neuronal cell death. The frequency of HEWL fibrillation was reduced significantly in the presence of proline and pro-AuNP coincubation, and two slightly different intermediate species were formed. The use of PEG-coated gold anthocyanins nanoparticles in neurodegenerative diseases could be a new therapeutic choice. Since the AuNPs SPR band amplitude is affected by Aβ40 amyloids, the intensity of the AuNPs SPR band is affected by Aβ40 amyloids. It is used to identify and measure the levels of Aβ40 amyloids, and it is also useful for characterizing the rate of Aβ amyloid formation.

## 6. Conclusions

Alzheimer’s disease is one of the most dangerous diseases facing humans, especially the elderly. Nowadays, nanotechnology plays an important role in treating several diseases. One of these diseases is Alzheimer’s disease. The usual traditional methods of treating Alzheimer’s disease sometimes fail to reach the cells that need treatment. Moreover, find it difficult to reach the brain cells, which weakens its effect on the accumulations of peptides causing the disease. However, advances in nanotechnology have led to the emergence of so-called nanoparticles, which can efficiently penetrate brain cells and deal with pathogens. This review provided a classification of those parts with a comprehensive explanation of the role of each. The classification was built into three categories, with an explanation of the characteristics of each category. This review sheds light on the role of each group in treating the disease and clarifies an important fact that nanoparticles have a promising future in treating such a dangerous disease.

## Figures and Tables

**Table 1:** Comparison between different types of nanoparticles used in Alzheimer’s disease treatment.

Classification	Name	Function and Merits	Average Diameter in nm	References
**Polymeric nanoparticles**	DGLs-PEG-RVG29-D-peptide/DNA NPs	It provides numerous reaction locations and good drug loading charges. Successful codelivery of blood-brain barrier-crossing drugs through brain-oriented ligand modifications was shown in vitro and in vivo.	97	[14]
	Drug-loaded PLGA nanoparticles	In transgenic AD mice, spatial memory and recognition were significantly improved	128.6	[32]
	PLGA@QT NPs	Low cytotoxicity when tested in vitro on SH-SY5Y cells	Between 100 and 150	[40]
	PGZ-NPs	The carriers received PGZ, which promoted 50 × higher brain endothelium absorption than free drugs and displayed a delayed in vitro release profile of PGZ.	155.0 ± 1.8	[33]
	Anti-A mAb-conjugated liposomes	Stable in serum protein incubation and in a position to bind to A in vitro monomers and fibrils	Between 124 and 134	[16]
	Poly(acrylic acid)-coated NPs	Abrogated Aβ aggregation at a sub-stoichiometric ratio of 1:2,000,000	8 and 18	[17]
	− PLGA NPs − PLGA NPs with OX26 mAb − PLGA NPs with OX26 mAb and DE2B4 mAb	The intake of immune nanoparticles with controlled peptide iA5 delivery without monoclonal antibody function was significantly increased.	153 ± 2 163 ± 3 166 ± 2	[35]
	PiB-MZF	It is stable, biocompatible. The relaxing rate of PiB-R2 MZF was 169.93 mM^−1^S^−1^, which showed great superparamagnetism as the negative T2 contrast agent. PiB-MZF also showed no cytotoxicity in two cell lines.	100	[43]
	NP(α−M)	Improving brain clearance in an LDLR-dependent way of 125I-radiolabeled Aβ1–42, reduction in Aβ deposition and reduced neuroinflammatory reactions.	94.26 ± 4.54	[20]
	Eu/GMP ICP	Has a self-adaptive property and rationally designing the competitive coordination interaction of Cu2þ between the guest CDs and Ab monomer.	From 40 to 50	[22]
	Se−Cur/PLGA	Cur-loaded Se-PLGA nanosphere drug delivery system will decrease the amyloid-β load in the brain samples of AD mice and healed the model mice’s memory deficit substantially.	160 ± 5	[37]
	CdS	The biologically synthesized PC-metal nanoparticles, in particular iron oxide, do not impact neuroblastoma cells’ viability.	50–60	[24]
	DBP-PLGA	Significantly inhibited Aβ aggregation in vitro. Moreover, intravenous injection of DBP-PLGA nanoparticles significantly attenuated the Aβ accumulation, neuroinflammation, neuronal loss and cognitive dysfunction in the 5XFAD mice.	226.6 ± 44.4 nm	[36]
	MEM–PEG–PLGA	Non-cytotoxic brain cell lines. Memantine adopted a slower release profile of NPs into the free medicine solution, minimizing the in vivo drug control frequency.	200	[38]
	PLGA-PEG-B6	Could tremendously improve the spatial learning and memory capability of APP/PS1 mice, compared with native Cur	Less 100	[42]
	Anti-Aβ1-42-NPs	Full memory defect correction; substantial decrease of the Aβ-soluble peptide and its brain oligomer level and significant increase of plasma Aβ levels.	182	[20]
	MoS2 QDs and TPP-MoS2 QDs	Exhibit a complete bifunctional nanozyme activity that prevents spontaneous neuroinflammation.	30 and 50	[30]
**Lipid nanoparticles**	S80−, PS−, and PA-functionalized SLNs	Could ameliorate the cognition impairment of rats more effectively than the conventional administration of nicotinamide.	112 ± 1.6, 124 ± 0.8, and 137 ± 1.05	[65]
	CN-SLNs	CN can be achieved therapeutically at lower doses and its oral bioavailability enhanced by encapsulating CN in SLNs.	240.0 ± 4.79	[67]
	TFB-SLNs	The therapeutic level of TFB could be transferred directly to the brain via the olfactory pathway, following the intranasal administration of polymers and lipid nanoparticles.	200	[68]
	SLN and NLC	NLC permeate more the blood–brain barrier, while amyloid-beta studies demonstrated NLC-transferrin has the capacity to inhibit fibril formation.	Lower than 250	[39]
	EPO-SLN	High potential for drug encapsulation and improved anti-colon cell efficacy	219.9 ± 15.6	[69]
	NR C NRb Cb	The positive charge of the coating formula ensured that particles were mucoadhesive and that they were prolonged in the nasal cavity.	335.76 ± 34.81 358.44 ± 25.89 419.47 ± 24.36 469.71 ± 49.07	[47]
	Span 60 and cholesterol	Used to solve the problem of the extensive rapid metabolism of rivastigmine.	100.7	[48]
	Solid lipid nanoparticles (SLN)	Their efficacy, user-friendliness, versatility and intellectual property opportunities through innovating drug delivery in particular for drug release shift systems	222 ± 21 to 414 ± 11	[71]
	APOE-DONSLN	ApoE, which binds BBB receptors, can be used to successfully target solid lipid nanoparticles	147.5 ± 0.8	[50]
	NLC	Low toxicity and toxicity against the cell line SH-SY5Y	Below 200	[75]
	RHT-SLNs	Improve the delivery of RHT brain targeting by producing and optimizing RHT-SLNs	15.6	[72]
	Lipid polymer hybrid NPs	Efficient, fast penetration into healthy albino rats of the bio-inspired surface-modified NPs	111.6 ± 11.4	[51]
	SLN and PLGA NPs	No toxicity, changes in body weight or clinical symptoms of the disease were found	200	[52]
	RT-loaded SLN	Zeta potential value of−10 mV was found, polydispersion index was found in the 0.3–0.6 range.	214	[73]
	RT loaded PLGA-Soya lecithin-Tween-80	Therapeutic prospect to treat AD and potential carrier for providing sustained brain delivery of RT	171.74	[34]
	Curcumin and meloxicam-loaded lipid-core nanocapsules (LNC)	No toxicity in relation to the parameters determined of all LNC evaluated in mice	424 nm (curcumin) and 365 nm (meloxicam)	[61]
	POPC: POPG 3:1	Characterization of simultaneous size and zeta potential in individual capillary nanoparticles and particle mixtures under physiological salinities.	76 ± 3	[58]
**Gold nanoparticles**	AuNPs	High functionality and high active area are used to improve the catalytic activity of captured AuNPs electrocatalytic tags.	20	[84]
	Chiral l− and d−glutathione (GSH) stabilized Au NPs	Can inhibit Aβ42 aggregation and cross BBB after intravenous administration without substantial toxicity.	3.3	[85]
	D−/L−Pe−Au	Major decrease in the cell index, indicating that cytotoxic effects on PC12 cells depend on concentration.	7	[93]
	GNPs	Therapeutic ability of GNPs with behavioral and oxidative stress parameters in GNP-treated mice	20	[98]
	GNPs	Clinical potential may suppress CNS inflammation and oxidative stress, alleviating secondary neurodegenerative processes and reserpine-induced neuronal cell death.	20	[99]
	Pro-AuNPs nbbAuNPs	HEWL fibrillation greatly reduced with proline and pro-AuNP coincubation, and two slightly different intermediate species were produced with these two systems as CD spectroscopy predicts.	529 nm and 523 nm	[100]
	PEG-coated AuNPs	PEG-coated gold anthocyanins nanoparticles may be a new therapeutic agent for neurodegenerative diseases	135 ± 5	[102]
	Citrate-based AuNPs	AuNPs SPR band intensity is susceptible to Aβ40 amyloids. This helps SPR test detect and semi-quantify Aβ40 amyloids and describe the kinetics of Aβ amyloid formation.	23	[107]

## Data Availability

Data is contained within the article.
